# Comparative Metabolome and Transcriptome Analyses of the Regulatory Mechanism of Light Intensity in the Synthesis of Endogenous Hormones and Anthocyanins in *Anoectochilus roxburghii* (Wall.) Lindl.

**DOI:** 10.3390/genes15080989

**Published:** 2024-07-26

**Authors:** Jiayu Cao, Jingjing Zeng, Ruoqun Hu, Wanfeng Liang, Tao Zheng, Junjie Yang, Xiaoying Liang, Xiaowei Huang, Ying Chen

**Affiliations:** 1 College of Landscape Architecture and Art, Fujian Agriculture and Forestry University, Fuzhou 350002, China; cjiayu2024@163.com (J.C.); 15528517916@163.com (J.Z.); 13169089371@163.com (R.H.); a576428098@163.com (W.L.); lxy12170222@163.com (X.L.); 18122667665@163.com (X.H.); 2 Fujian Institute of Tropical Crops, Zhangzhou 363001, China; 18659699110@163.com (T.Z.); junjieyang202309@163.com (J.Y.)

**Keywords:** transcription factor, promoter cis-acting element, RNA-seq, phytohormone, qRT-PCR validation, correlation analysis

## Abstract

To explore the regulatory mechanism of endogenous hormones in the synthesis of anthocyanins in *Anoectochilus roxburghii* (Wall.) Lindl (*A. roxburghii*) under different light intensities, this study used metabolomics and transcriptomics techniques to identify the key genes and transcription factors involved in anthocyanin biosynthesis. We also analyzed the changes in and correlations between plant endogenous hormones and anthocyanin metabolites under different light intensities. The results indicate that light intensity significantly affects the levels of anthocyanin glycosides and endogenous hormones in leaves. A total of 38 anthocyanin-related differential metabolites were identified. Under 75% light transmittance (T3 treatment), the leaves exhibited the highest anthocyanin content and differentially expressed genes such as chalcone synthase (*CHS*), flavonol synthase (*FLS*), and flavonoid 3′-monooxygenase (*F3*′*H*) exhibited the highest expression levels. Additionally, 13 transcription factors were found to have regulatory relationships with 7 enzyme genes, with 11 possessing cis-elements responsive to plant hormones. The expression of six genes and two transcription factors was validated using qRT-PCR, with the results agreeing with those obtained using RNA sequencing. This study revealed that by modulating endogenous hormones and transcription factors, light intensity plays a pivotal role in regulating anthocyanin glycoside synthesis in *A. roxburghii* leaves. These findings provide insights into the molecular mechanisms underlying light-induced changes in leaf coloration and contribute to our knowledge of plant secondary metabolite regulation caused by environmental factors.

## 1. Introduction

*A. roxburghii* (Wall.) Lindl is a perennial herb of the genus Epipactis in the Orchidaceae family and is named for the golden yellow or orange-red reticulate venation on the upper surface of its leaves. With its delicate plant form and elegant leaf shape, it is a valued indoor ornamental plant. *A. roxburghii* is mainly distributed in China, including Fujian, Zhejiang, Jiangxi, and Guizhou provinces; Japan; Sri Lanka; India; and Nepal [1]. This species has traditionally been considered one of the most valuable medicinal plants. Many years ago, *A. roxburghii* was listed as a protected species in the Convention on International Trade in Endangered Species of Wild Fauna and Flora (CITES). Because *A. roxburghii* is widely used in medicine, health care, beauty, drinking products, and many other areas, its market demand as a medicinal plant has increased yearly [1]. However, the market for *A. roxburghii* as an ornamental plant remains to be developed. The hue of medicinal plants is closely tied to anthocyanin biosynthesis and content levels. Therefore, investigating the biosynthetic mechanism of anthocyanins in *A. roxburghii* leaves is important for regulating leaf color and the market development of this species as an ornamental plant.

Anthocyanins are among the most significant plant pigments derived from flavonoid metabolism, and their diverse types contribute to the richness of plant colors. They typically accumulate in flowers, fruits, seeds, and leaves of the angiosperms, providing red, purple, and blue plant hues [2]. Depending on the degree of hydroxylation and methylation, anthocyanins can be classified into six major groups: pelargonidin, cyanidin, delphinidin, peonidin, petudinin, and malvidin glycosides [3]. Cyanidin and pelargonidin glycosides are often found in red-colored leaves, such as *Acer rubrum* L. [4] and *Camellia japonica* L. [5], while delphinidin glycosides, malvidin, peonidin, and petudinin glycosides are primarily present in deep blue and purple plant organs, such as *Muscari botryoides* (L.) Mill [6] and *Rhododendron pulchrum* sweet (Ericaceae) [7]. The biosynthesis and regulation of anthocyanins are accomplished through the combined effects of intrinsic genetic backgrounds and extrinsic environmental factors mediated by a complex regulatory network [8]. The anthocyanin synthesis is regulated by two types of genes: structural genes, which encode various enzymes required in the metabolic pathway, and regulatory genes, which encode transcription factors that control the spatiotemporal expression levels of the structural genes. The structural genes include two significant groups, namely, the upstream gene group (*CHS*, *CHI*, and *F3H*) and the downstream gene group (*F3’H*, *F3*′*5*′*H*, *DFR*, *ANS*, and *UFGT*). Regulatory genes primarily consist of those that encode the MYB, bHLH, and WD40 transcription factors, which regulate the expression of genes related to anthocyanin biosynthesis by binding to corresponding cis-acting elements in the promoters of structural genes [9]. Research on the biosynthesis pathways of anthocyanins in various plant organs has reached a highly advanced state. Yet, there are few reports on the synthesis mechanism of anthocyanins in *A. roxburghii* leaves.

The environment in which plants exist has a profound influence on the synthesis of anthocyanins, with light being one of the most significant influencing factors. The relationship between light intensity and anthocyanin biosynthesis is a crucial research direction in horticultural breeding and cultivation. Light can regulate the expression levels of genes involved in anthocyanin synthesis pathways and structural genes [10], enhancing enzyme activity to promote the synthesis and accumulation of anthocyanins in plants. Researchers have screened differentially expressed genes and metabolites in *Brassica napus* L. seedlings under various light conditions. They found that the color changes in *B. napus* L. organs and tissues induced by strong light were associated with anthocyanin accumulation and changes in the expression of related genes, which guided tissue research on *B. napus* L. seedlings [11]. Zhao used combined transcriptomic and metabolomic analysis to identify the nutritional differences in *Fagopyrum tataricum* (L.) Gaertn. under various light conditions and the impact of light on the anthocyanin synthesis pathway, unearthing 12 anthocyanin-related structural genes [12]. Nakatsuka used a combined analysis of transcriptional metabolism to discover that the flower color of *Lilium brownii* var. *viridulum* Baker lightens, anthocyanin content decreases, and bifunctional dihydroflavonol 4-reductase (*DFR*) gene expression down-regulates after shading [13]. However, there is currently limited research on the regulatory mechanism of anthocyanin synthesis in *A. roxburghii* in response to changes in light intensity. Therefore, using light to regulate its anthocyanins has become an important objective for enhancing the ornamental value of *A. roxburghii*.

Studying plant hormones has emerged as a frontier and popular field in contemporary international plant science. Based on current research findings, plant hormones can be categorized into nine major classes, namely, auxins (AUXs), cytokinins (CKs), gibberellins (GAs), abscisic acid (ABA), ethylene (ETH), jasmonic acid (JA), salicylic acid (SA), brassinosteroids (BRs), and strigolactones (SLs) [14]. Anthocyanin biosynthesis is regulated by endogenous plant hormones [15], which act on regulatory genes to control structural genes, ultimately leading to anthocyanin accumulation. For instance, ethylene inhibits the transcription of the *SlAN2-like* gene in tomatoes (*Lycopersicon esculentum* Mill.), suppressing the biosynthesis of anthocyanins [16]; treatment with gibberellin induces the expression of the *Ipomoea nil* (L.) Roth. *CHS* gene, promoting anthocyanin accumulation [17]; the anthocyanin content in grape (*Vitis vinifera* L.) skins treated with salicylic acid generally exhibits an initial decrease followed by an increase, unlike in untreated groups, where it increases [18]. However, there is still no research on the leaf coloration mechanism under different light intensities or the endogenous hormone regulation of *A. roxburghii*.

To explore the mechanism of anthocyanin biosynthesis and the role of closely related endogenous hormones in *A. roxburghii* leaves under different light intensities, this study used metabolomics and transcriptomics techniques and bioinformatics methods to achieve the following goals: to analyze the transcriptional regulation mechanism underlying the specific accumulation of secondary metabolites; to identify the key genes and transcription factors of anthocyanin biosynthesis under different light intensities; to analyze the changes in and correlation between endogenous hormones and anthocyanin metabolites; and to construct a regulatory network of these genes and metabolites. The results of this study may provide a theoretical basis and practical guidance for hormone regulation in *A. roxburghii* leaf color production.

## 2. Materials and Methods

### 2.1. Treatment of Experimental Materials

*A. roxburghii* tissue culture seedlings were provided by the experimental base of Fujian Agriculture and Forestry University (Fujian Institute of Tropical Crops, Zhangzhou, China, 117.31° E, 24.38° N) in 2023. The experiment was conducted in the greenhouse of the Tropical Crops Research Institute of Fujian Province from August 2023 to December 2023. The average yearly temperature was 21.1 °C, with a lowest temperature of 25 °C and a highest temperature of 33 °C from August to October. The greenhouse was kept at a temperature range of 23 °C to 25 °C, with a humidity of 75% to 85%, an average light intensity of 120 μmol/(m^2^·s), and a photoperiod of 12 h by using a water curtain, fan, and spray system.

We selected the *A. roxburghii* bottle seedlings with a growth stage of about 3 months, a plant height of 8–10 cm, 2–3 roots, and a stem diameter of about 0.25 cm, using a 5 × 5 arrangement with 5 rows of 5 bottles per treatment, for a total of 25 bottles. Both sides of the bottle seedlings were protected plants, and the middle bottle seedlings were selected as experimental materials. Different shading nets (2, 4, and 8 needles) were used under natural light intensity in the greenhouse. The shading nets were not removed during the whole leaf growth period, and the upper layer of the middle test bottle seedlings was selected for sampling. Shading net T1 treatment: 8 stitches, light transmittance of 25%, average light intensity of 25 μmol/(m^2^·s). Shading net T2 treatment: 4 stitches, light transmittance of 50%, average light intensity of 50 μmol/(m^2^·s). Shading net T3 treatment: 2 stitches, light transmittance of 75%, average light intensity of 80 μmol/(m^2^·s). Each treatment was set up with 3 replicates and 5 bottles for each replicate. The sampling period was 15 days. The leaves were taken from the same direction as *A. roxburghii* plants, frozen with liquid nitrogen, stored in a −80 °C refrigerator, and sent to Beijing BMK Biotechnology (Beijing, China) for metabolomics and transcriptomics analyses and the quantitative real-time PCR (qRT-PCR) analysis. The management measures for all bottle seedlings at the test site were consistent.

### 2.2. Determination of Metabolome

The leaves of *A. roxburghii* were vacuum freeze-dried and then ground into a powder. A 50 mg sample of the leaves was weighed, and 1000 μL of an extraction solution containing an internal standard (methanol:acetonitrile:water = 2:2:1, internal standard concentration 20 mg/L) was added. The solution was vortex mixed for 30 s, followed by the addition of steel balls and treatment with a 45 Hz grinding instrument (POWTEQ, Beijing Grinder Instrument Co., Ltd., Beijing, China) for 10 min [19]. Subsequently, it underwent ultrasonication for 10 min (in an ice-water bath). The liquid chromatography-mass spectrometry (LC-MS) system for metabolomics analysis is composed of Waters Acquity I-Class PLUS ultra-high performance liquid tandem Waters (Shanghai, China) Xevo G2-XS QTof high-resolution mass spectrometer. The column used is purchased from Waters (Shanghai, China) Acquity ultra-performance liquid chromatography–tandem (UPLC) HSS T3 column (1.8 um 2.1 × 100 mm). The Kyoto Encyclopedia of Genes and Genomes (KEGG) database (http://www.genome.jp/kegg/, accessed on 17 February 2024) was employed to annotate the differential metabolites of *A. roxburghii* leaves under different light intensities and to screen for target metabolites for analysis.

### 2.3. Determination of the Transcriptome

Bowtie2 (version 1.3.3) software [20] was used to compare the clean reading with Universal Gene (UniGene) (https://ncbiinsights.ncbi.nlm.nih.gov/tag/unigene/, accessed on 17 February 2024), and RSEM (version 1.3.3) software [21] was used to calculate the gene expression of each sample. Combined with the KEGG annotation results, the genes related to anthocyanin biosynthesis in the transcriptome database were mined. Based on the count value of genes in each sample, differential analysis software was used to screen differentially expressed genes, and the DESeq R software [22] package (1.10.1) was used to analyze the differential expression of the sample group. Genes with adjusted *p* values < 0.05 found by deseq were designated as differentially expressed. The *p*-value was corrected by the false discovery rate (FDR). The differential multiple |log2 foldchange| (FC) threshold value ≥ 2 or ≤0.5 and a FDR < 0.01 were selected to further screen the target differentially expressed genes. The data have been registered in the NCBI Sequence Read Archive (SRA) database (https://trace.ncbi.nlm.nih.gov/Traces/sra/, accessed on 22 June 2024) under the GenBank accession numbers SRR29496896, SRR29496895, SRR29496894, SRR29496893, SRR29496892, SRR29496891, SRR29496890, SRR29496889, SRR29496888, SRR29496887, SRR29496886, and SRR29496885. The electropherograms presenting the RNA bands in agarose gels (Figures S1–S9) and the RNA integrity number (Figure S10) were in the Supplementary Materials.

### 2.4. Joint Analysis of the Transcriptome and Metabolome

Referring to the method of JI. [23], the differential genes and metabolites were annotated on the KEGG synthesis pathway map (http://www.kegg.jp/kegg/pathway.html, accessed on 17 February 2024) of target metabolites for further analysis. The Pearson correlation analysis was performed on the expression of anthocyanin-related differential genes and the quantitative value of the anthocyanin glycoside score using IBM SPSS Statistics 26 (SPSS) software. The results of Pearson’s correlation coefficient (PCC) values > 0.9 and *p* < 0.05 were selected, and the interaction network diagram was drawn by Cytoscape 3.10.2 software.

### 2.5. Correlation Analysis between Endogenous Hormones and Genes of Differential Enzymes in the Anthocyanin Biosynthesis Pathway

To further investigate the correlation between the levels of endogenous plant hormones and the expression of differential enzyme genes involved in anthocyanin synthesis pathways, we selected data with a PCC > 0.9 and *p* < 0.05 for correlation analysis and used Cytoscape 3.10.2 software to construct a corresponding “plant hormone-enzyme gene expression” correlation network [23].

### 2.6. Correlation Analysis of Endogenous Hormones and Anthocyanin Metabolites in Plants

To investigate the correlation between differential endogenous plant hormones and differential anthocyanin metabolites in *A. roxburghii* leaves under different light treatments, Pearson correlation coefficients were used to determine the correlation between the relative content of differential metabolites and plant hormones. Based on the threshold value (correlation coefficient PCC > 0.9, *p* < 0.05), screening was performed, and the “plant hormone-anthocyanin metabolite” correlation network was constructed using Cytoscape 3.10.2 software [24].

### 2.7. Prediction of Transcription Factors

The transcription factor prediction tool on the PlantTFDB (https://planttfdb.gao-lab.org/, accessed on 17 February 2024) website was used to predict the transcription factors of all gene sequences in *A. roxburghii*. Then the transcription factor families were classified and statistically analyzed using Excel (Excel 2023) tools. Pearson correlation analysis (correlation coefficient PCC > 0.9, *p* < 0.05) was used to analyze the transcriptional regulation relationship between transcription factors and enzyme genes [25] and further construct a possible “transcription factor-enzyme gene expression” regulatory network using Cytoscape 3.10.2 software.

### 2.8. Analysis of Cis-Acting Elements in Transcription Factor Promoters

To analyze the possible regulatory mechanisms of transcription factors associated with anthocyanin glycoside biosynthesis in *A. roxburghii* leaves in response to phytohormones. The corresponding transcripts were obtained through the Phytozome database (https://phytozome-next.jgi.doe.gov/, accessed on 17 February 2024) to obtain the nucleic acid sequences 2000 bp upstream of the transcription start sites of the corresponding transcription factors. Then the promoter cis-elements of the transcription factors were analyzed by PlantCARE (http://bioinformatics.Psb.ugent.be/webtools/plantcare/html/, accessed on 17 February 2024), and finally, the transcripts were visualized by using the TBtools 2.102 for visual mapping [26].

### 2.9. Real-Time Quantitative PCR Verification

Six genes—flavonol synthase (*FLS-2*), chalcone synthase (*CHS-3*, *CHS-4*, *CHS-7*), flavonoid 3′-monooxygenase (*F3*′*H-3*), and bifunctional dihydroflavonol 4-reductase (*DFR*)—and two transcription factors—bHLH130 and ERF066—were validated with qRT-PCR using the RT^2^ SYBR Green ROX qPCR Mastermix Kit (Qiagen, Beijing, China) and using the *Actin* gene as the internal reference gene. All the qRT-PCR analyses were performed using the following conditions: denaturation at 95 °C for 3 min, followed by 40 cycles of 95 °C for 10 s, and then at 60 °C for 30 s. All components were configured according to the qPCR reaction system, followed by centrifugation at 6000 rpm for 30 s at 4 °C on a PCR plate centrifuge. Subsequently, the plate was placed in a quantitative PCR instrument for amplification and melt curve analysis (60~95 °C, +1 °C/cycle, holding time 4 s). The relative expression levels of the key enzyme genes and transcription factor genes were calculated using the 2^−ΔΔCt^ method [27], and each gene was subjected to three biological replicates. The base sequences are shown in Table 1. The GenBank accession of the reference gene is JF825424, the amplification and melt curves are shown in the Supplementary Materials (Figures S11–S28).

## 3. Results

### 3.1. Analysis of Metabolomic Differences

#### 3.1.1. Analysis of Differential Anthocyanin Glycoside Metabolites

**Figure 1:**
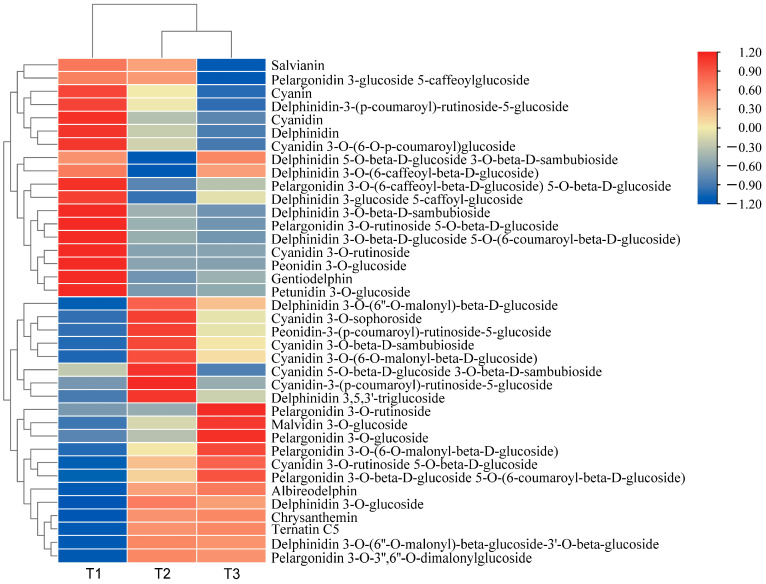
Heat map of the clustering of differential anthocyanin glycoside metabolites in *A. roxburghii* (Wall.) Lindl leaves at different light intensities.

By selecting for variable importance in projection (VIP) ≥ 1, fold change ≥ 2, and fold change ≤ 0.5, differential metabolites related to anthocyanin synthesis were screened. A total of 35 flavonoid-related differential metabolites and 38 differential anthocyanin metabolites were screened. The relative differential anthocyanin glycoside contents were clustered, and a heat map was drawn based on their relative contents (Figure 1). The color block distribution distinguishes *A. roxburghii* leaves under different light treatments, and the specific differences are shown in Table 2.

In the comparison between T1 (with light transmittance of 25%) and T3 (with light transmittance of 75%), a total of 30 distinct anthocyanin metabolites were identified. Among these, 16 metabolites were up-regulated, and 14 were down-regulated. Compared with T1, the content of the 16 metabolites in T3 significantly increased, including pelargonidin 3-*O*-3″, 6″-*O*-dimalonylglucoside, chrysanthemin, and delphinidin 3-*O*-(6″-*O*-malonyl)-β-D-glucoside. We speculated that delphinidin, cyanidin, and pelargonidin glycosides are the primary metabolites responsible for leaf coloration of the leaves. In T1, delphinidin 3 and 3′-di-glucoside 5-(6-caffeoyl) glucoside were hardly detected, whereas they increased by 37.30 times in T3. This suggests that delphinidin 3 and 3′-di-glucoside 5-(6-caffeoyl) glucoside may be the key metabolites responsible for the color variation in *A. roxburghii* induced by light.

Between T1 and T2 (with light transmittance of 50%), 34 differential anthocyanin metabolites were identified. Of these, the content of 18 differential metabolites was significantly up-regulated, and the content of 16 was significantly down-regulated. Compared with T1 vs. T3, the contents of peonidin-3-(p-coumaroyl)-rutinoside-5-glucoside, delphinidin 3-*O*-glucoside, and delphinidin 3,5,3′-triglucoside significantly increased in T1 vs. T2, but there were no significant differences in T1 vs. T3. We speculated that a light transmittance of 50% is more favorable for accumulating these three metabolites.

In the comparison between T2 and T3, 25 differential metabolites were identified, of which 9 were up-regulated and 16 were down-regulated. Compared with the up-regulated metabolites in T1 vs. T3, there were seven down-regulated metabolites in T2 vs. T3, including peonidin-3-p-coumaroyl-rutinoside-5-glucoside and cyanidin-3-(p-coumaroyl)-rutinoside-5-glucoside. We speculated that a certain degree of shading may be more conducive to accumulating these metabolites, while excessively high light intensity may inhibit their synthesis. The content of five metabolites, including malvidin 3-*O*-glucoside and cyanidin 3-*O*-rutinoside 5-*O*-β-d-glucoside, increased across all three comparative groups. We speculated that the increased light intensity may benefit the accumulation of these metabolites.

Compared with the other treatments, the anthocyanin content was higher in T3, and the leaf color was more pronounced. We speculated that accumulating anthocyanins contributes to the vivid appearance of the leaves.

#### 3.1.2. Analysis of Differential Endogenous Hormone Metabolites

**Figure 2:**
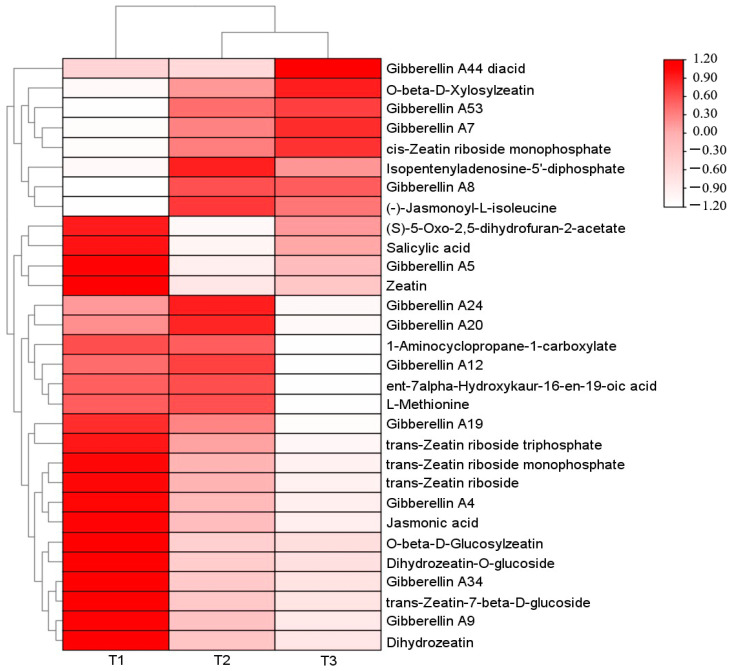
Heat map of clustering of different phytohormone metabolites in *A. roxburghii* leaves under different light intensities.

A total of 29 differential metabolites related to plant endogenous hormones were screened among the three comparative groups. Among them, 24 differential metabolites were screened in T1 (with light transmittance of 25%) vs. T2 (with light transmittance of 50%) (8 up-regulated and 16 down-regulated), 22 differential metabolites were screened in T2 vs. T3 (with light transmittance of 75%) (7 up-regulated and 15 down-regulated), and 27 differential metabolites were screened in T1 vs. T3 (7 up-regulated and 20 down-regulated). A heat map was drawn based on their relative content (Figure 2).

A total of 13 differential metabolites were screened during the synthesis of gibberellins (GA), including 11 types of GA, with the exception of Gibberellin A7 (GA7), Gibberellin A53 (GA53), Gibberellin A8 (GA8), and Gibberellin A44 (GA44) diacid (the precursor substance of GA), which had the highest content under T3 treatment (with light transmittance of 75%). The content of the other identified GA metabolites was higher under T1 treatment than T3. A total of one differential metabolite was screened during the synthesis of salicylic acid (SA), with the highest content under T1 treatment and the lowest content under T3 treatment. A total of 11 different metabolites were screened in the synthesis pathway of zeatin (ZT), among which *O*-β-d-xylosylzeatin, isopentenyladenosine-5′-diphosphate, and cis-zeatin ribose side monophosphate had the highest content in T3, and other zeatin-related metabolites had the highest content in T1. On the synthesis pathways of jasmonic acid (JA) and ethylene (ETH), two differential metabolites were screened, and their content showed a downward trend with the increase in light intensity. The specific metabolite profile is shown in Table 3.

### 3.2. Transcriptome Differential Gene Expression Analysis

To study the expression of enzymes related to the anthocyanin synthesis pathway in *A. roxburghii* leaves under different light intensities, the genes related to flavonoid biosynthesis and the anthocyanin synthesis pathway were screened, in total, 66 flavonoid biosynthesis-related sequences and 2 anthocyanin synthesis-related sequences were annotated. With a fold change ≥ 2 and a false discovery rate (FDR) < 0.01 as the criteria, further differential screening of anthocyanin synthesis-related genes in *A. roxburghii* leaves under different light intensities was performed, showing that these genes were differentially expressed only in the flavonoid synthesis pathway.

**Figure 3:**
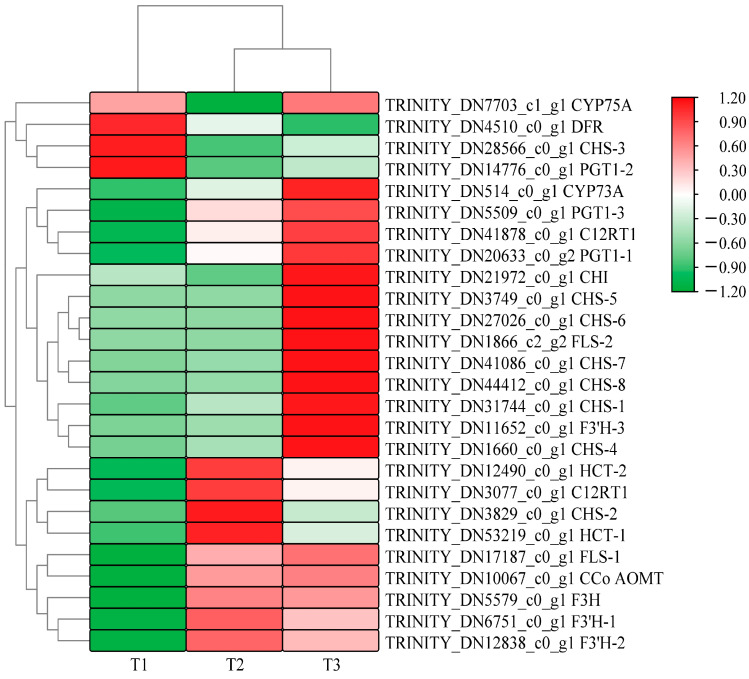
Heat map of clustering of differential genes related to anthocyanins glycoside synthesis in *A. roxburghii* leaves at different light intensities.

Figure 3 shows that *A. roxburghii* leaves treated with different light intensities can be distinguished by their color block distribution. Many highly expressed genes were enriched in the T2 (with light transmittance of 50%) and T3 treatments (with light transmittance of 75%), but most genes had relatively low expression levels in the T1 treatment (with light transmittance of 25%). A total of 26 differentially expressed sequences for flavonoid biosynthesis were obtained under different light intensities: 14 existed in T1 vs. T2, 12 existed in T2 vs. T3, and 19 existed in T1 vs. T3. The specific differences are shown in Table 4, Table 5 and Table 6. In the upstream pathway of flavonoid/anthocyanin biosynthesis, some differentially expressed genes were identified in the comparisons of T1 vs. T2, T2 vs. T3, and T1 vs. T3, including chalcone synthase (*CHS*), chalcone isomerase (*CHI*), flavanone-3-hydroxylase (*F3H*), flavonol synthase (*FLS*), etc. In the three comparison groups, several genes had their highest expression levels in T3, including one *FLS* gene, six *CHS* genes, and one flavonoid 3′-monooxygenase (*F3*′*H-3*) gene, indicating that increased light intensity comprehensively promoted the overall metabolism of flavonoids in leaves.

Compared with T1, *F3H* (TRINITY_DN5579_c0_g1), *FLS* (TRINITY_DN17187_c0_g1), *F3*′*H* (TRINITY_DN12838_c0_g1), *PGT1* (TRINITY_DN20633_c0_g2), *CCo AOMT* (TRINITY_DN10067_c0_g1), and *CYP75B1* (TRINITY_DN6751_c0_g1) showed higher gene expression levels in T3 and T2 treatments, but the differences were not significant in T2 vs. T3. *CHS* (TRINITY_DN31744_c0_g1 and TRINITY_DN1660_c0_g1) was significantly up-regulated in the three comparison groups. Notably, the transcription levels of *F3*′*H-3* (TRINITY_DN12838_c0_g1, TRINITY_DN11652_c0_g1) were significantly higher than the expression levels of other structural genes in the whole anthocyanin synthesis pathway. These results suggest that *CHS*, *F3*′*H*, and *FLS* may be the key genes regulating the metabolism of flavonoids and anthocyanins in *A. roxburghii* leaves in response to light intensity.

### 3.3. Combined Analysis of Transcriptome and Metabolome

**Figure 4:**
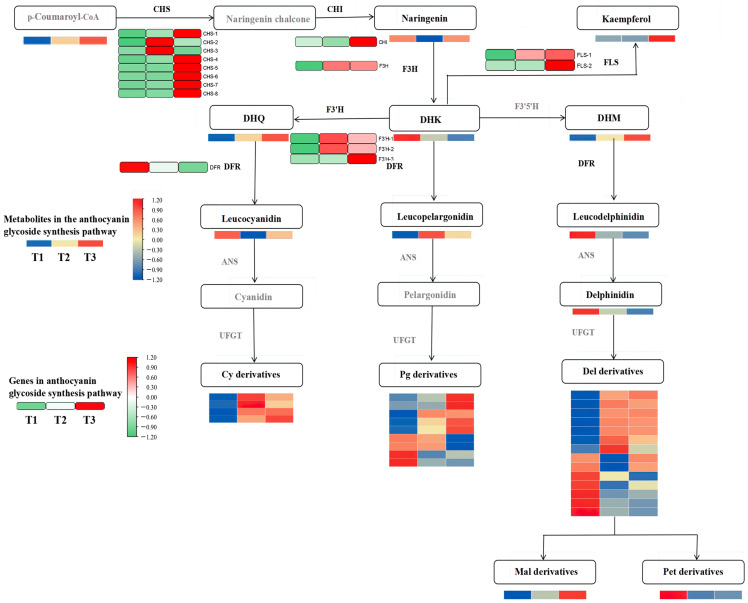
Conjoint analysis of transcriptome metabolism of anthocyanins in *A. roxburghii* leaves under different light intensities.

Differential metabolites and genes were jointly mapped onto the anthocyanin pathway. Figure 4 shows that the contents of dihydroquercetin, cyanidin 3-glucoside, delphinidin 3-glucoside, and pelargonidin 3-glucoside were significantly up-regulated in the flavonoid synthesis pathway. Notably, the pelargonidin 3-glucoside content was significantly higher than in other metabolites.

The differentially expressed genes encoded by the key enzymes in the anthocyanin synthesis pathway, including chalcone synthase (*CHS*), flavonol synthase (*FLS*), flavonoid 3-hydroxylase (*F3H* and *F3*′*H*), and bifunctional dihydroflavonol 4-reductase (*DFR*), are associated with the production of metabolic products and catalyze the synthesis of flavonoids and anthocyanins in the phenylpropane pathway. *CHS* is the initial gene in the upstream pathway of flavonoid synthesis and plays a significant role in the overall content of anthocyanins. Apart from *CHS-2* and *CHS-3*, most of the other *CHS* genes, as well as the chalcone isomerase (*CHI*) gene, exhibited the most significant expression levels in the T3 treatment (with light transmittance of 75%), which catalyzed the formation of naringenin chalcone from p-coumaroyl-CoA, leading to naringenin synthesis. *F3H* is the first key gene in the pelargonidin synthesis pathway and showed a significantly higher expression level in T3 than in T1 (with light transmittance of 25%). This may explain why pelargonidin 3-glucoside content increases with increasing light intensity. The *F3H* gene catalyzes the formation of naringenin into dihydrokaempferol. Notably, the dihydrokaempferol content was the lowest in T3, which was inconsistent with the expression of the *F3H* gene. This discrepancy could be attributed to the significant expression of the *FLS* and *F3*′*H* genes in T3. *FLS* further catalyzes dihydrokaempferol to synthesize other flavonol compounds, and the *F3*′*H* catalyzes dihydrokaempferol to form dihydroquercetin.

The content of leucoanthocyanins—precursors for anthocyanin synthesis—was jointly regulated by *FLS* and *DFR*. *FLS* and *DFR* competed for dihydroflavonol substrates. The expression level of the *DFR* gene was highest in the T1 treatment and lowest in the T3 treatment, and we speculated that its catalytic capacity for dihydroflavonol substrates diminished in T3, which was negatively correlated with the pattern of anthocyanin glycoside accumulation. In T3, the expression of *FLS* genes reversed, with their catalytic capacity for dihydroflavonols increasing with an increase in light intensity. The *F3*′*H* genes are pivotal in the anthocyanin synthesis pathway, and they displayed elevated expression levels in response to increasing light intensity. Particularly, the expression of *F3*′*H-3* genes was the most significant, mirroring the accumulation patterns of delphinidin and cyanidin glycosides in *A. roxburghii* leaves under the T3 treatment.

In general, the highest anthocyanin content in *A. roxburghii* leaves under the T3 treatment may primarily stem from the regulatory role of the *CHS* gene upstream of flavonoid synthesis. *CHS* gene expression was significantly up-regulated under the T3 treatment, and many flavonoid compounds were synthesized, further increasing the anthocyanin content in the leaves. Genes closely related to flavonoid synthesis and anthocyanin metabolism downstream include *FLS-1*, *FLS-2*, *F3*′*H-3*, and *DFR*. Even though the gene expression of *DFR* and *FLS* negatively affected anthocyanin synthesis, the total anthocyanin content in leaves under the T3 treatment was still significantly higher than under the T1 treatment because dihydroflavonol substrate content was significantly higher than under the T1 treatment.

### 3.4. Correlation between Hormone Levels and Transcript Abundance of Anthocyanin Biosynthesis in A. roxburghii

**Figure 5:**
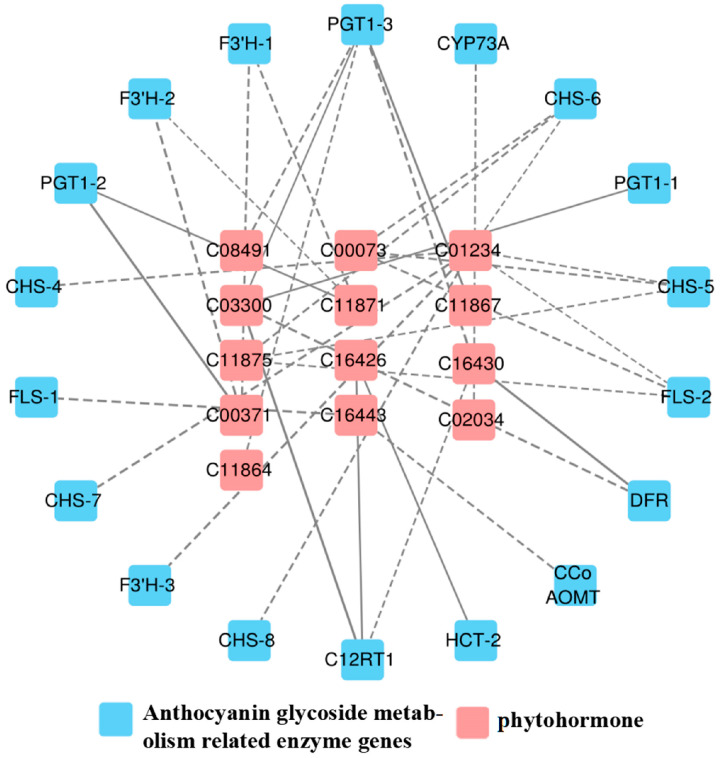
Correlation network of “phytohormone–anthocyanin glycoside genes” in *A. roxburghii* leaves at different light intensities. The solid line indicates positive regulation, the dashed line indicates negative regulation, and the thicker the line, the stronger the correlation of regulation.

As shown in Figure 5, a total of 13 differential hormones and 18 anthocyanin differential genes were associated, including 2 flavonol synthase (*FLS*) genes, 1 trans-cinnamate 4-monooxygenase (*CYP73A*) gene, 3 phlorizin synthase (*PGT1*) genes, 3 flavonoid 3′-monooxygenase (*F3*′*H*) genes, 5 chalcone synthase (*CHS*) genes, 1 flavanone 7-*O*-glucoside 2″-*O*-β-L-rhamnosyltransferase (*C12RT1*) gene, 1 shikimate *O*-hydroxycinnamoyltransferase (*HCT*) gene, 1 caffeoyl-CoA *O*-methyltransferase (*CCo AOMT*) gene, and 1 bifunctional dihydroflavonol 4-reductase (*DFR*) gene.

In the 34 generated correlation analyses, 25 showed negative coefficients of correlation, while the rest were positive. Among these, genes such as Gibberellin A7 (GA7), Gibberellin A5 (GA5), *O*-β-d-xylosylzeatin, and *PGT1* genes exhibited positive correlations; the trans-zeatin riboside monophosphate and *DFR* showed a positive correlation; Isopentenyladenosine-5′-diphosphate was positively correlated with *C12RT1* and *HCT*; and *O*-β-d-xylosylzeatin demonstrated a positive correlation with *C12RT1*. Otherwise, all other correlation coefficients indicated negative correlations, among which 1-aminocyclopropane-1-carboxylate (ACC) and *F3*′*H-3* exhibit a strong negative correlation, as shown in Table 7.

### 3.5. Correlation between Hormone Levels and Anthocyanin Glycoside Metabolite Content in A. roxburghii

**Figure 6:**
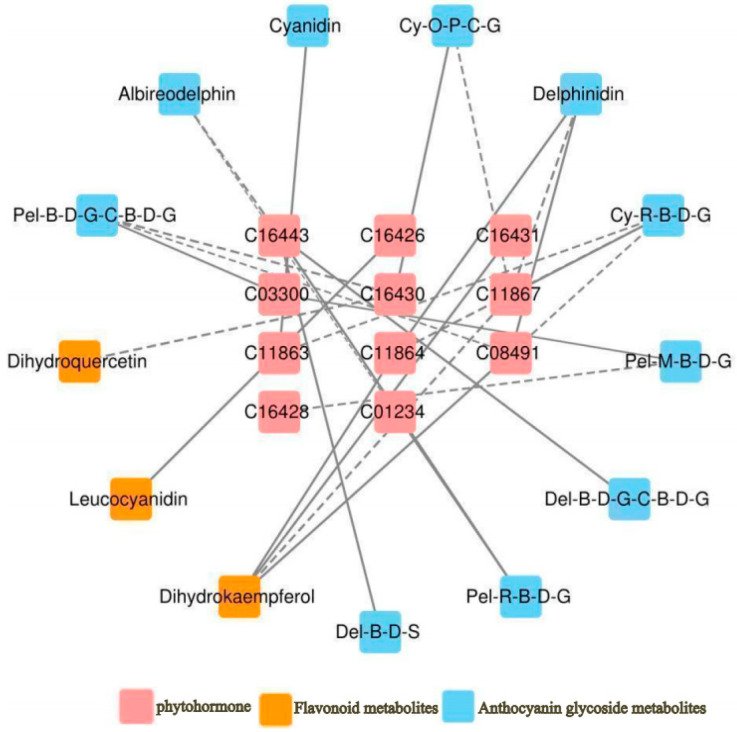
Correlation network of “phytohormone–anthocyanidin glycoside–related metabolites” in *A. roxburghii* leaves at different light intensities. The solid line indicates positive regulation, the dashed line indicates negative regulation, and the thicker the line, the stronger the correlation of regulation.

As shown in Figure 6, a total of 11 hormones were correlated with 10 anthocyanin glycosides and 3 flavonoid glycosides. Among these, Gibberellin A7 (GA7) was negatively correlated with delphinidin, dihydrokaempferol, and two cyanidin glycosides; Gibberellin A9 (GA9) was positively correlated with cyanidin; and Gibberellin A4 (GA4) was negatively correlated with cyanidin 3-*O*-rutinoside 5-*O*-β-d-glucoside and positively correlated with dihydrokaempferol. trans-zeatin-7-β-d-glucoside was negatively correlated with delphinidin 3-*O*-β-d-glucoside 5-*O*-(6-coumaroyl-β-d-glucoside) and positively correlated with delphinidin 3-*O*-β-d-sambubioside; trans-zeatin riboside monophosphate was negatively correlated with pelargonidin 3-*O*-β-d-glucoside 5-*O*-(6-coumaroyl-β-d-glucoside) and dihydroquercetin. 1-aminocyclopropane-1-carboxylate (ACC) was negatively correlated with pelargonidin 3-*O*-β-d-glucoside 5-*O*-(6-coumaroyl-β-d-glucoside), isopentenyladenosine-5′-diphosphate was positively correlated with leucocyanidin, jasmonic acid was positively correlated with delphinidin. The specific correlation coefficients are presented in Table 8.

### 3.6. Prediction and Analysis of the Regulatory Relationship between Endogenous Plant Hormones and Anthocyanin Metabolite Biosynthesis Enzyme Genes in A. roxburghii Using Transcription Factors

In the transcriptome data of *A. roxburghii* leaves treated with different light intensities, 1422 genes were predicted to encode 55 transcription factor families. The top 10 transcription factor families were MYB_related (33), FAR1 (32), ERF (32), C2H2 (32), bHLH (28), NAC (25), GRAS (21), bZIP (19), WRKY (17), and Dof (15). The results are shown in Table 9.

**Figure 7:**
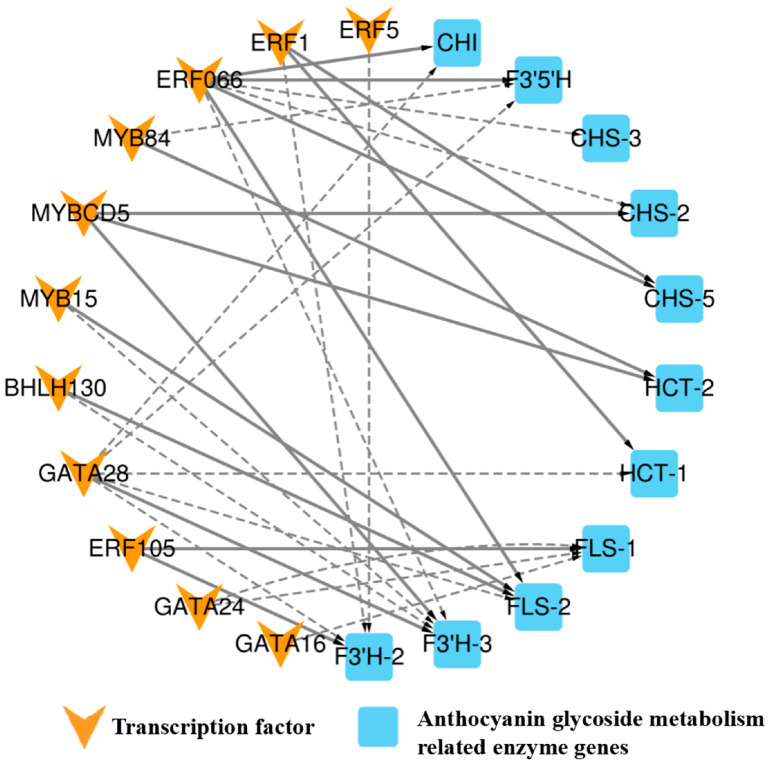
Regulatory network diagram of “transcription factor–anthocyanin metabolism-related enzyme gene expression” in *A. roxburghii* leaves under different light intensities. The solid line indicates positive regulation, the dashed line indicates negative regulation, and the thicker the line, the stronger the correlation of regulation.

To further investigate the key transcription factors involved in regulating anthocyanin metabolism in *A. roxburghii* leaves under various light-intensity treatments, we employed Pearson correlation analysis to identify data with a correlation coefficient greater than 90% between the transcription factors and enzyme genes. In conjunction with the PlantTFDB database, we conducted regulation predictions for the six transcription factor families, MYB, bHLH, GATA, ERF, TCP, and bZIP, and constructed a regulatory network diagram for “transcription factor–enzyme gene expression”. Figure 7 shows the regulatory relationship between 13 transcription factors and 7 enzymes.

The figure shows that BHLH30 positively regulated flavonol synthase (*FLS-2*) and negatively regulated the flavonoid 3′-monooxygenase (*F3*′*H-3*) enzyme gene. MYBCD5 was involved in positively regulating the *F3*′*H-3*, shikimate *O*-hydroxycinnamoyltransferase (*HCT-2*), and chalcone synthase (*CHS-2*) enzyme genes, while MYB15 negatively regulated *F3*′*H-3* and positively regulated the *FLS-2* enzyme gene. MYB84 was positively regulated *HCT-2* and negatively regulated flavonoid 3′5′-hydroxylase (*F3*′*5*′*H*) enzyme gene. ERF066 negatively regulated the *F3*′*H-3*, *CHS-3*, and *CHS-2* enzyme genes and positively regulated the *FLS-2*, *CHI*, *CHS-5*, and *F3*′*5*′*H* enzyme genes. ERF5 negatively regulated the *F3*′*H-2* enzyme gene, while ERF105 positively regulated the *F3*′*H-2* and *FLS-1* genes. GATA28 negatively regulated the *FLS-2*, *CHI*, *HCT-1*, *F3*′*5*′*H*, and *F3*′*H-2* genes and positively regulated the *F3*′*H-3* gene. GATA16 negatively regulated the *FLS-1* gene. TCP19 positively regulated the *DFR* gene, and bZIP21 positively regulated the *F3*′*H-1* gene. ERF1 positively regulated the *CHS-5* and *HCT-1* genes and negatively regulated the *F3*′*H-2* gene. GATA24 negatively regulated the *FLS-1* gene. The coexpression patterns of anthocyanin-related transcription factors and enzyme genes are summarized in Table 10.

To further investigate the regulatory mechanism of endogenous hormones in anthocyanin metabolism-related key transcription factors in *A. roxburghii* leaves under different light-intensity treatments, we conducted promoter cis-element analysis on the predicted transcription factors, selecting elements responsive to plant hormones, including the methyl jasmonate response element (G-box/CGTCA-motif), the abscisic acid response element (ABRE9), the salicylic acid response element (TCA-element), the gibberellic acid response element (TATC-box/P-box), and the auxin response element (AuxRR-core).

**Figure 8:**
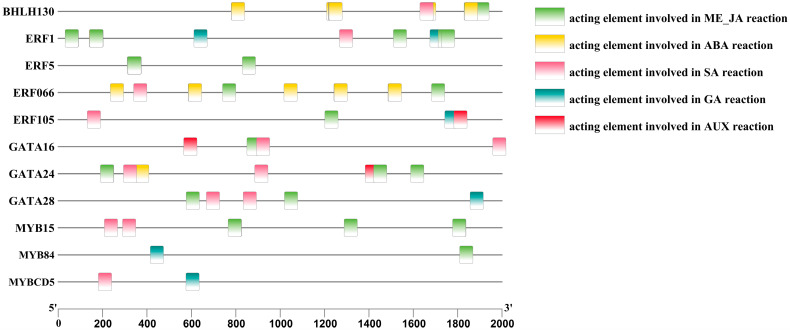
Analysis of the promoter cis-elements of anthocyanin-related transcription factors in the leaves of *A. roxburghii* under different light intensities.

Figure 8 shows that, among the 13 transcription factors that regulate anthocyanin metabolism-related enzymes, 11 had cis-acting elements that respond to plant hormones. ERF066, MYB15, and BHLH30 were regulated by three plant hormones: salicylic acid (SA), abscisic acid (ABA), and methyl jasmonate (Me_JA). ERF1 was regulated by four plant hormones: SA, Gibberellins (GAs), ABA, and Me_JA. ERF5 was regulated by one plant hormone: Me_JA. ERF105 was regulated by four plant hormones: SA, GA, auxin (AUX), and Me_JA. GATA16 was regulated by three plant hormones: AUX, SA, and Me_JA. GATA24 was regulated by four plant hormones: SA, ABA, Me_JA, and AUX. GATA28 was regulated by three plant hormones: Me_JA, SA, and GA. MYB84 was regulated by two plant hormones: Me_JA and GA. MYBCD5 was regulated by two plant hormones: SA and GA. The promoter status of anthocyanin-related transcription factors in response to plant hormones is shown in Table 11.

To further explore the relationship between plant hormones and transcription factors in *A. roxburghii* under different light-intensity treatments and identify potential regulatory factors related to anthocyanin accumulation and metabolism, we determined the relationship between plant hormone content and transcription factor expression using the Pearson correlation coefficient. We selected data with a correlation coefficient of Pearson’s correlation coefficient (PCC) > 0.9 and *p* < 0.05 for analysis.

Ent-7alpha-hydroxykaur-16-en-19-oic acid was negatively correlated with BHLH130 and MYB15, while Gibberellin A53 (GA53) was negatively correlated with NHLH74. Gibberellin A24 (GA24) and Gibberellin A20 (GA20) were positively correlated with MYBCD5 and GATA1, while GA8 was negatively correlated with GATA24. GA44 diacid was positively correlated with BHLH130 and MYB15, Gibberellin A34 (GA34) was positively correlated with BHLH74 and BHLH78, and SA was positively correlated with ERF5. Dihydrozeatin was positively correlated with BHLH74; zeatin was negatively correlated with ERF12; trans-zeatin-7-β-d-glucoside was positively correlated with BHLH8, BHLH74, and BHLH78; and trans-zeatin riboside monophosphate was positively correlated with TCP19 and bZIP21. Isopentenyladenosine-5′-diphosphate was negatively correlated with ERF5, and *O*-β-d-glucosylzeatin and dihydrozeatin-*O*-glucoside were positively correlated with BHLH8, BHLH74, and BHLH78, and negatively correlated with ERF105. Trans-zeatin riboside triphosphate was positively correlated with TCP19 and bZIP21, while *O*-β-D-Xylosylzeatin was negatively correlated with TCP19 and bZIP21. (-)-jasmonoyl-l-isoleucine was positively correlated with ERF12, jasmonic acid was positively correlated with TCP19, L-methionine was negatively correlated with BHLH130 and MYB15, 1-aminocyclopropane-1-carboxylate (ACC) was negatively correlated with BHLH130 and MYB15, and positively correlated with GATA28. The specific correlation coefficients are shown in Table 12.

### 3.7. qRT-PCR Verification of Key Transcription Factors and Enzyme Genes in A. roxburghii Leaves under Different Light Intensities

**Figure 9:**
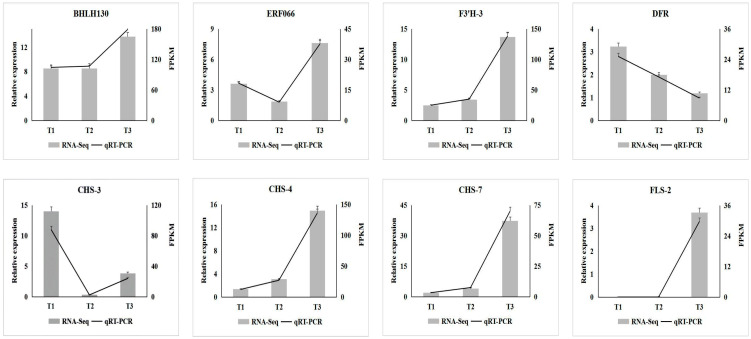
qRT-PCR analysis of differential enzymes and transcription factors in *A. roxburghii* leaves under different light intensity treatments.

To further verify the expression of key transcription factors and enzyme genes involved in anthocyanin biosynthesis in *A. roxburghii* under different intensity treatments, eight genes (including two transcription factor genes and six enzyme genes) were selected from the screened key genes for qRT-PCR detection. As shown in Figure 9, the expression levels of transcription factor genes BHLH130 (TRINITY_DN221_c1_g1), *FLS-2* (TRINITY_DN1866_c2_g2), *CHS-7* (TRINITY_DN41086_c0_g1), *CHS-4* (TRINITY_DN1660_c0_g1) and *F3*′*H-3* (TRINITY_DN11652_c0_g1) involved in anthocyanin biosynthesis gradually increased with the increase in light intensity. With the increase in light intensity, the expression level of ERF066 (TRINITY_DN7304_c0_g1) and *CHS-3* (TRINITY_DN28566_c0_g1) first decreased and then increased, while the expression level of *DFR* (TRINITY_DN4510_c0_g1) gradually decreased. The expression of the eight genes in *A. roxburghii* leaves under different light-intensity treatments obtained by quantitative analysis through qRT-PCR was consistent with the results of transcriptome sequencing.

## 4. Discussion

### 4.1. Light Intensity Directly Promotes Anthocyanin Synthesis by Regulating the Content of Endogenous Hormones

The metabolome analysis showed that the relative content of anthocyanins in the leaves was highest in T3 (with light transmittance of 75%), while the content and types of anthocyanins in the leaves were the lowest in T1 (with light transmittance of 25%). We speculated that a light transmittance of 75% was more conducive to accumulating anthocyanins in *A. roxburghii* leaves. The authors of [15] determined the anthocyanin content of *Vaccinium uliginosum* L. under four different light intensities. With increased light intensity, the anthocyanin content significantly increased, and the content of anthocyanin metabolites under 75% light intensity was 1.09 to 4.08 times that of other light-intensity treatments. This is consistent with the results of this study, indicating that appropriate light can significantly promote the synthesis of anthocyanins and increase anthocyanin content in leaves. By measuring the anthocyanin compounds in *A. roxburghii* under different light intensities, we found that pelargonidin, delphinidin, cyanidin, and malvidin may be the main anthocyanins responsible for the differences in leaf color, similar to the results of Li’s study on the detection of anthocyanin components in the light-induced O’Neil blueberry variety (*Vaccinium corymbosum* L.) [26]. This was the main reason for the color deepening during the growth and development of this blueberry variety.

The endogenous phytohormone difference analysis showed that a total of 30 metabolites related to plant endogenous hormones (including jasmonic acid, salicylic acid, ethylene, zeatin, and gibberellins) were identified in the leaves under different light intensities. We observed that most of the gibberellin, salicylic acid, zeatin, jasmonic acid, and ethylene had the highest content under the treatment of 25% light transmittance, and their levels gradually decreased with increasing light intensity, indicating that shading is more conducive to accumulating plant hormones. In *oilseed peony* leaves, mild, moderate, and severe shading increases Indole acetic acid concentrations by 38.1%, 45.5%, and 49.0%, respectively, while gibberellin A3 concentrations rise by 6.3%, 7.6%, and 11.7% [28]. In *Camellia sinensis*, the content of gibberellin and ethylene decreases with increasing light intensity [29]. This is consistent with the results of the present study, indicating that weaker light is more conducive to accumulating endogenous hormones in plants.

Research has found that the synthesis of anthocyanins is largely influenced by various plant hormones, such as gibberellin, cytokinins, and ethylene [30]. However, there are few studies on whether there is an intrinsic relationship between endogenous plant hormones and the differential distribution of anthocyanins in *A. roxburghii* under different light-intensity treatments. Therefore, we attempted to construct a preliminary “plant hormone–anthocyanin metabolite” correlation network under different light-intensity treatments through Pearson correlation analysis. The results showed 11 hormones correlate with 10 anthocyanin metabolites and 3 flavonoid metabolites. Most gibberellins have different regulatory effects on anthocyanins owing to different types and concentrations. Gibberellin A7 was negatively correlated with delphinidin, dihydrokaempferol, and two cyanidin glycosides; Gibberellin A9 was positively correlated with cyanidin; and ACC was negatively correlated with pelargonidin 3-O-β-d-glucoside 5-O-(6-coumaroyl-β-d-glucoside). We discovered that the levels of these phytohormones and anthocyanin metabolites exhibit either a positive or negative correlation under different light intensities, and we speculate that an intrinsic regulatory mechanism may exist between them. Gao [31] discovered that under different exogenous gibberellin treatment conditions, there were variations in the peak contents of quercetin, kaempferol, and anthocyanin in *Ginkgo biloba* leaves; Ahmad [32] discovered that the accumulation of flavonoids (quercetin, kaempferol) in the *Talinum paniculatum* exhibits a peak under different concentrations of salicylic acid treatment; Macarena [33] found that using an exogenous ethylene treatment on “Honeycrisp” apples (*Malus pumila*) resulted in the largest increase in anthocyanin biosynthesis genes and total anthocyanins.

In summary, many studies have confirmed the intrinsic regulatory relationship between plant hormones and anthocyanin metabolites. Therefore, we can try to regulate the content of endogenous plant hormones in *A. roxburghii* and thereby affect the content of highly related anthocyanin metabolites to highly accumulate target metabolites in the leaves of *A. roxburghii* and regulate its leaf color changes. However, its intrinsic regulatory mechanism needs further research.

### 4.2. Endogenous Hormones Regulate the Synthesis of Anthocyanin Metabolites by Regulating the Expression of Key Enzyme Genes

Through transcriptome data analysis, we found that synthesizing anthocyanins under different light-intensity treatments may be mainly affected by flavonoid 3′-monooxygenase (*F3*′*H*), chalcone synthase (*CHS*), flavonol synthase (*FLS*), and bifunctional dihydroflavonol 4-reductase (*DFR*). The expression of the *F3H* gene significantly increased under the T3 treatment (75% transmittance), indicating that the *F3H* gene is involved in biosynthesizing related pigments, which has also been confirmed in flower color research on *Impatiens uliginosa*. The expression level of the *F3H* gene was the highest in the dark red color at the beginning of flowering and lowest in the pink color at the peak of flowering [34]. The *F3*′*H* gene may be expressed in various plant tissues. The up-regulated expression of the *F3*′*H* gene often causes leaves to turn red, while down-regulated expression reduces the total anthocyanin content [35]; its main role is catalyzing the synthesis of dihydroquercetin from dihydrokaempferol. The gene expression of the *F3*′*H* enzyme was also high under the T3 treatment, which may have led to an increase in *F3*′*H* enzyme activity and a decrease in dihydrokaempferol content, consistent with previous research results. The enzymes encoded by the *CHS* and *CHI* genes catalyze p-coumaroyl-C*O*A and naringenin-chalcone, respectively, which form dihydrokaempferol, an important precursor for anthocyanin synthesis. The expression of the *CHS* and *CHI* genes was consistent with the pattern of anthocyanin accumulation, suggesting that they may affect anthocyanin synthesis in *A. roxburghii* leaves.

Plant hormones are important signaling molecules in plants and regulate (promote or inhibit) their own physiological processes under certain conditions. Previous studies have shown that plant hormones can regulate anthocyanin synthesis by affecting structural genes related to anthocyanin synthesis [36]. However, the changes in hormones and related mechanisms of anthocyanin biosynthesis in *A. roxburghii* were unknown. Therefore, we analyzed the differences in endogenous plant hormones in *A. roxburghii* under different light treatments and attempted to analyze the relationship between plant hormones and anthocyanin-related enzyme genes in *A. roxburghii* leaves by constructing a plant hormone and enzyme gene expression network.

The correlation analysis of endogenous hormone and enzyme gene expression in *A. roxburghii* leaves treated with different light intensities showed that the precursor of ethylene synthesis, ACC, was negatively correlated with the downstream *FLS* and *CHS* genes involved in anthocyanin synthesis and strongly negatively correlated with the *F3*′*H* gene. Many studies have shown that ethylene plays a negative regulatory role in anthocyanin synthesis. For example, Ni [37] found that ethylene has a significant inhibitory effect on the biosynthesis of *Pyrus* spp. anthocyanins, similar to the way ACC acted in this study, which was also why the *Pyrus* spp. flowers in this study were lighter colors. As early as 1971, Craker [38] studied the effect of ethylene treatment on anthocyanin synthesis in Sorghum bicolor during induction and found that ethylene can promote anthocyanin accumulation in the early lag phase while inhibiting anthocyanin accumulation in the late lag phase. In dark conditions, ethylene can inhibit the accumulation of anthocyanins in the kernel pericarp. When treated with the ethylene synthesis inhibitor (S) trans-2-amino-4-(2-aminoethoxy)-3-butenoic acid, it was found that the expression levels of anthocyanin synthesis-related genes such as *CHS*, *ANS*, and *F3H* were significantly up-regulated [39]. In addition, the regulation pattern of gibberellin on genes in the anthocyanin synthesis pathway is very diverse. Gibberellin A5 is negatively correlated with the upstream gene F3′H in the anthocyanin synthesis pathway and positively correlated with Phlorizin synthase, while gibberellin A7 is positively correlated with Phlorizin synthase, which may be related to the type and concentration of gibberellin. Wang [40] found that the anthocyanin content in *Oryza sativa* L. with a low nitrogen treatment was significantly higher than in the wild type, and exogenous gibberellin (GA3) treatment inhibited the accumulation of anthocyanin. Further research found that low-nitrogen treatments could inhibit the production of gibberellins (GA1, GA3, and GA4) and promote anthocyanin accumulation. Gibberellin 2-oxidase (GA2ox) plays an important role in the gibberellin metabolic pathway. Wang [41] found that OsGA2ox8 can indirectly regulate anthocyanin genes to participate in the anthocyanin synthesis regulation process. In summary, endogenous hormones can regulate the synthesis of anthocyanin metabolites by regulating the expression of key enzyme genes.

### 4.3. Endogenous Hormones Regulate Enzyme Genes by Activating Transcription Factors, Thereby Promoting the Synthesis of Anthocyanin Metabolites

In the actual anthocyanin synthesis process, transcription factors (TFs) also participate in the relevant regulation. TFs can regulate metabolism by activating or inhibiting the expression of enzyme genes [42], while plant hormones can not only regulate the expression of enzyme genes but also affect the accumulation of anthocyanins by regulating the expression of transcription factor genes [43]. Therefore, this study further constructed a regulatory “plant hormones, transcription factors, enzyme gene expression, and anthocyanin accumulation” network and explored the possible regulatory relationship between plant hormones and transcription factors, as well as between transcription factors and enzyme gene expression from the perspective of transcription factor regulation. The results show that 13 transcription factors that regulate anthocyanin metabolism-related enzymes and 7 enzymes have regulatory relationships in *A. roxburghii* leaves under different light treatments, of which 11 transcription factors have cis-elements responding to plant hormones.

The transcription factor ERF5 negatively regulated the *F3*′*H-2* enzyme gene, while ERF105 positively regulated the *F3*′*H-2* and *FLS-1* genes. We speculated that ERF-type transcription factors in *A. roxburghii* leaves do not have a single mode of regulating anthocyanin glycosides. Research shows that hormones can regulate anthocyanin glycoside synthesis in plants through ERF. For example, ethylene accelerates anthocyanin accumulation by promoting the transcription of Md MYB1 and key genes for anthocyanin synthesis, while Md MYB1 induces the transcription of ethylene-responsive factor Md ERF3 to further enhance ethylene-mediated anthocyanin accumulation and *M. pumila* Mill. fruit coloring [44]. In the jasmonic acid-regulated anthocyanin synthesis pathway mediated by Md ERF1B, the interaction between the jasmonic acid signaling pathway inhibitor Md JAZ5/10 and the Md ERF1B protein significantly reduces the activation of the Md MYC2 promoter by Md ERF1B, inhibiting anthocyanin accumulation [45]. GATA transcription factors can respond to multiple hormone signaling molecules and light stimulation, regulating plant growth and development. In this study, GATA28 negatively regulated *FLS-2*, *CHI*, *HCT-1*, *F3*′*5*′*H*, and *F3*′*H-2*, while GATA16 and GATAG24 negatively regulated the *FLS-1* gene. In the leaves of *A. roxburghii* treated with different light treatments, we found that GATA-type transcription factors negatively regulated the genes of enzymes related to anthocyanin synthesis. There are few studies on regulating anthocyanin glycosides with GATA transcription factors. However, Liu [46] analyzed the cis-acting elements of GATA transcription factors in *Morus alba* L. and found that GATAs may be involved in transcriptional regulation and plant hormone signaling transduction under biotic and abiotic stress. This study shows that the transcription factors involved in anthocyanin biosynthesis pathways in *A. roxburghii* may respond to stimulating plant hormones such as salicylic acid, gibberellins, abscisic acid, and methyl jasmonate, regulating the expression of key enzyme genes in their respective synthesis pathways and, thus, playing a role in the “plant hormones, transcription factors, and enzyme gene expression” regulatory network.

In general, the darker leaf color under 75% light transmittance in *A. roxburghii* leaves with different light treatments may be due to the stimulation of three key transcription factors, BHLH130, GATA28, and ERF066, by plant hormones such as ethylene. These hormones regulate the expression of four enzyme genes, *FLS-2*, *CHS-4*, *F3*′*H-3*, and *DFR*, thereby affecting the accumulation of metabolites such as delphinidin 3,3′-diglucoside 5-(6-caffeoyl)glucoside, malvidin 3-*O*-glucoside, pelargonidin 3-*O*-β-d-glucoside 5-*O*-(6-coumaroyl-β-d-glucoside), pelargonidin 3-*O*-(6-*O*-malonyl-β-d-glucoside), and cyanidin 3-*O*-rutinoside 5-*O*-β-d-glucoside, which change the leaf color.

## 5. Conclusions

In this study, we conducted a correlation analysis between metabolites, transcription factors, key genes, and endogenous hormones, indicating the potential regulatory role of hormones in anthocyanin metabolism. A 75% light transmittance value is most conducive to anthocyanin synthesis in *A. roxburghii*. Under the influence of light, hormone signaling activates downstream transcription factors, and these activated transcription factors further regulate the expression of relevant enzyme genes for anthocyanin glycoside metabolism, affecting the accumulation of anthocyanin metabolites and forming a mechanism by which different light intensities regulate the changes in *A. roxburghii* leaf color. This study provides a reference for further studies of the regulation of plant secondary metabolism accumulation by light environmental factors and new insights and data for the role of the molecular regulatory network of plant hormones in anthocyanin synthesis.

## Figures and Tables

**Table 1:** Primer sequence information of the key enzyme genes and transcription factors validated by qRT-PCR.

Gene Name	Forward Primer Sequence (5′-3′)	Reverse Primer Sequence (5′-3′)
*Actin 1*	AAGCTGTTCTTTCCCTATATGCTAGTGG	CTTCTCCTTGATGTCCCTGACAATTT
*FLS-2*	ATTGCACCTTCCGAGTCTGG	AGCTATCTCCTTCCGGCCAT
*CHS-3*	TCATGCATATGCCTCCAATCT	TTCCAGACACGTGCTAGCTG
*CHS-4*	AACCTGTGGAACTCGTGCAT	GATTTGAGCTTGTGGCCGTC
*CHS-7*	CACTCCTTGATGGCCTTGGT	GGCCAGAAGCAACCAAACTT
*F3*′*H-3*	TTCATCTTCCTCACCACGCC	CCTCACCAAACCCTCCACTC
*DFR*	CTGACCTCAAGAGAGGCACG	ATGCACGAGTTCCACAGGTT
BHLH130	TCCTGGCTTCATGGCTCATC	GAGCCTCTCCTTTGCACCTT
ERF066	GACCCTACTCGCAGGATTCG	AGTCGTACCCACCGGATACA

**Table 2:** Difference of Anthocyanidin glycosides in *A. roxburghii* leaves under different light treatments.

Compound	T1_vs_T2_Regulated	T2_vs_T3_Regulated	T1_vs_T3_Regulated	Formula	KEGG_Annotation
Delphinidin 3-*O*-(6″-*O*-malonyl)-β-D-glucoside	up	down	up	C_24_H_23_O_15_	C16301
Ternatin C5	up	unchanged	up	C_36_H_42_O_25_	C16303
Albireodelphin	up	unchanged	up	C_42_H_47_O_25_	C16354
Pelargonidin 3-*O*-3″, 6″-*O*-dimalonyl-glucoside	up	unchanged	up	C_27_H_25_O_16_	C16288
Delphinidin 3-*O*-(6″-*O*-malonyl)-β-glucoside-3′-*O*-β-glucoside	up	unchanged	up	C_30_H_33_O_20_	C16304
Pelargonidin 3-*O*-β-D-glucoside 5-*O*-(6-coumaroyl-β-D-glucoside)	up	up	up	C_36_H_37_O_17_	C16349
Cyanidin 3-*O*-(6-*O*-malonyl-β-D-glucoside)	up	down	up	C_24_H_23_O_14_	C12643
Pelargonidin 3-*O*-glucoside	-	up	up	C_21_H_21_O_10_	C12137
Chrysanthemin	up	unchanged	up	C_21_H_21_O_11_	C08604
Malvidin 3-*O*-glucoside	up	up	up	C_23_H_25_O_12_	C12140
Pelargonidin 3-*O*-(6-*O*-malonyl-β-D-glucoside)	up	up	up	C_24_H_23_O_13_	C12642
Cyanidin 3-*O*-rutinoside 5-*O*-β-D-glucoside	up	up	up	C_33_H_41_O_20_	C12646
Cyanidin 3-*O*-sophoroside	up	unchanged	up	C_27_H_31_O_16_	C16306
Cyanidin 3-*O*-β-D-sambubioside	up	down	up	C_26_H_29_O_15_	C20490
Pelargonidin 3-*O*-rutinoside	unchanged	up	up	C_27_H_31_O_14_	C12644
Cyanidin-3-(p-coumaroyl)-rutinoside-5-glucoside	up	down	up	C_42_H_47_O_22_	C16292
Delphinidin 3, 5, 3′-triglucoside	up	down	unchanged	C_33_H_41_O_22_	C16314
Delphinidin 3-*O*-glucoside	up	unchanged	unchanged	C_21_H_21_O_12_	C12138
Peonidin-3-(p-coumaroyl)-rutinoside-5-glucoside	up	down	unchanged	C_43_H_49_O_22_	C16293
Delphinidin 5-*O*-β-D-glucoside 3-*O*-β-D-sambubioside	down	up	unchanged	C_32_H_39_O_21_	C20494
Delphinidin 3-*O*-(6-caffeoyl-β-D-glucoside)	down	up	unchanged	C_30_H_27_O_15_	C16367
Delphinidin 3-glucoside 5-caffoyl-glucoside	down	unchanged	unchanged	C_36_H_37_O_20_	C16313
Cyanidin 5-*O*-β-D-glucoside 3-*O*-β-D-sambubioside	up	down	unchanged	C_32_H_39_O_20_	C20493
Pelargonidin 3-glucoside 5-caffeoyl-glucoside	unchanged	down	unchanged	C_36_H_37_O_18_	C16371
Delphinidin	down	down	down	C_15_H_10_O_7_	C05908
Cyanidin 3-*O*-rutinoside	down	unchanged	down	C_27_H_31_O_15_	C08620
Pelargonidin 3-*O*-(6-caffeoyl-β-D-glucoside) 5-*O*-β-D-glucoside	down	up	down	C_36_H_37_O_18_	C12640
Cyanidin 3-*O*-(6-*O*-p-coumaroyl)-glucoside	down	down	down	C_30_H_27_O_13_	C12095
Gentiodelphin	down	unchanged	down	C_51_H_53_O_28_	C08641
Salvianin	unchanged	down	down	C_42_H_41_O_24_	C12647
Pelargonidin 3-*O*-rutinoside 5-*O*-β-D-glucoside	down	down	down	C_33_H_41_O_19_	C12645
Peonidin 3-*O*-glucoside	down	unchanged	down	C_22_H_23_O_11_	C12141
Cyanidin	down	down	down	C_15_H_10_O_6_	C05905
Petunidin 3-*O*-glucoside	down	unchanged	down	C_22_H_23_O_12_	C12139
Cyanin	down	down	down	C_27_H_30_O_16_	C08639
Delphinidin 3-*O*-β-D-glucoside 5-*O*-(6-coumaroyl-β-D-glucoside)	down	down	down	C_36_H_37_O_19_	C16351
Delphinidin-3-(p-coumaroyl)-rutinoside-5-glucoside	down	down	down	C_42_H_47_O_23_	C16294
Delphinidin 3-*O*-β-D-sambubioside	down	down	down	C_26_H_29_O_16_	C20491

**Table 3:** Differences in hormone metabolites in *A. roxburghii* leaves under different light treatments.

Hormone Classification	Compound	T1_vs_T2_Regulated	T2_vs_T3_Regulated	T1_vs_T3_Regulated
GA	ent-7alpha-Hydroxykaur-16-en-19-oic acid	unchanged	down	down
	Gibberellin A12	unchanged	down	down
	Gibberellin A19	down	down	down
	Gibberellin A7	up	up	up
	Gibberellin A9	down	down	down
	Gibberellin A5	down	up	down
	Gibberellin A53	up	unchanged	up
	Gibberellin A24	up	down	down
	Gibberellin A20	up	down	down
	Gibberellin A8	up	unchanged	up
	Gibberellin A44 diacid	unchanged	up	up
	Gibberellin A34	down	unchanged	down
	Gibberellin A4	down	down	down
SA	Salicylic acid	down	up	unchanged
ZT	Dihydrozeatin	down	unchanged	down
	Zeatin	down	up	down
	trans-Zeatin-7-β-D-glucoside	down	down	down
	trans-Zeatin riboside monophosphate	down	down	down
	Isopentenyladenosine-5′-diphosphate	up	down	up
	*O*-β-D-Glucosylzeatin	down	unchanged	down
	Dihydrozeatin-*O*-glucoside	down	unchanged	down
	trans-Zeatin riboside	down	down	down
	trans-Zeatin riboside triphosphate	unchanged	unchanged	down
	cis-Zeatin riboside monophosphate	up	up	up
	*O*-β-D-Xylosylzeatin	up	up	up
JA	(-)-Jasmonoyl-L-isoleucine	unchanged	down	unchanged
	Jasmonic acid	down	down	down
ETH	L-Methionine	down	down	down
	1-Aminocyclopropane-1-carboxylate	down	down	down

**Table 4:** T1 vs. T2 differential Anthocyanidin glycosidase gene.

Gene Function	Unigene	Corresponding Enzyme	log2FC (T1 vs. T2)	Gene Annotation	Enzyme KO
*CCo AOMT*	TRINITY_DN10067_c0_g1	[EC:2.1.1.104]	1.98	Caffeoyl-CoA *O*-methyltransferase	K00588
*F3*′*H-1*	TRINITY_DN6751_c0_g1	[EC:1.14.14.82]	1.88	Flavonoid 3′-monooxygenase	K05280
*F3*′*H-2*	TRINITY_DN12838_c0_g1	[EC:1.14.14.82]	1.5	Flavonoid 3′-monooxygenase	K05280
*CHS-1*	TRINITY_DN31744_c0_g1	[EC:2.3.1.74]	1.76	Chalcone synthase	K00660
*CHS-2*	TRINITY_DN3829_c0_g1	[EC:2.3.1.74]	1.5	Chalcone synthase	K00660
*CHS-3*	TRINITY_DN28566_c0_g1	[EC:2.3.1.74]	−2.43	Chalcone synthase	K00660
*CHS-4*	TRINITY_DN1660_c0_g1	[EC:2.3.1.74]	1.27	Chalcone synthase	K00660
*FLS-1*	TRINITY_DN17187_c0_g1	[EC:1.14.20.6]	1.3	Flavonol synthase	K05278
*PGT1-1*	TRINITY_DN20633_c0_g2	[EC:2.4.1.357]	1.23	Phlorizin synthase	K22845
*PGT1-2*	TRINITY_DN14776_c0_g1	[EC:2.4.1.357]	−2.29	Phlorizin synthase	K22845
*C12RT1*	TRINITY_DN3077_c0_g1	[EC:2.4.1.236]	1.16	Flavanone 7-*O*-glucoside 2″-*O*-β-L-rhamnosyltransferase	K13080
*HCT-1*	TRINITY_DN53219_c0_g1	[EC:2.3.1.133]	1.11	Shikimate *O*-hydroxycinnamoyltransferase	K13065
*F3H*	TRINITY_DN5579_c0_g1	[EC:1.14.11.9]	1	Flavanone-3-hydroxylase	K00475
*CYP75A*	TRINITY_DN7703_c1_g1	[EC:1.14.14.81]	−2.95	flavonoid 3′, 5′-hydroxylase	K13083

**Table 5:** T2 vs. T3 differential Anthocyanidin glycosidase gene.

Gene Function	Unigene	Corresponding Enzyme	log2FC (T2 vs. T3)	Gene Annotation	Enzyme KO
*FLS-2*	TRINITY_DN1866_c2_g2	[EC:1.14.20.6]	10.37	Flavonol synthase	K05278
*CHS-5*	TRINITY_DN3749_c0_g1	[EC:2.3.1.74]	5.57	Chalcone synthase	K00660
*CHS-6*	TRINITY_DN27026_c0_g1	[EC:2.3.1.74]	4.78	Chalcone synthase	K00660
*CHS-7*	TRINITY_DN41086_c0_g1	[EC:2.3.1.74]	3.19	Chalcone synthase	K00660
*CHS-8*	TRINITY_DN44412_c0_g1	[EC:2.3.1.74]	2.99	Chalcone synthase	K00660
*CHS-4*	TRINITY_DN1660_c0_g1	[EC:2.3.1.74]	2.52	Chalcone synthase	K00660
*CHS-1*	TRINITY_DN31744_c0_g1	[EC:2.3.1.74]	2.06	Chalcone synthase	K00660
*CHS-3*	TRINITY_DN28566_c0_g1	[EC:2.3.1.74]	1.42	Chalcone synthase	K00660
*CYP75A*	TRINITY_DN7703_c1_g1	[EC:1.14.14.81]	3.47	Flavonoid 3′, 5′-hydroxylase	K13083
*F3*′*H-3*	TRINITY_DN11652_c0_g1	[EC:1.14.14.82]	2.14	Flavonoid 3′-monooxygenase	K05280
*CHI*	TRINITY_DN21972_c0_g1	[EC:5.5.1.6]	1.22	Chalcone isomerase	K01859
*HCT-2*	TRINITY_DN12490_c0_g1	[EC:2.3.1.133]	−1.28	Shikimate *O*-hydroxycinnamoyltransferase	K13065

**Table 6:** T1 vs. T3 differential Anthocyanidin glycosidase gene.

Gene Function	Unigene	Corresponding Enzyme	log2FC (T1 vs. T3)	Gene Annotation	Enzyme KO
*FLS-2*	TRINITY_DN1866_c2_g2	[EC:1.14.20.6]	11.24	Flavonol synthase	K05278
*FLS-1*	TRINITY_DN17187_c0_g1	[EC:1.14.20.6]	1.5	Flavonol synthase	K05278
*CHS-3*	TRINITY_DN28566_c0_g1	[EC:2.3.1.74]	−1.42	Chalcone synthase	K00660
*CHS-5*	TRINITY_DN3749_c0_g1	[EC:2.3.1.74]	5.57	Chalcone synthase	K00660
*CHS-6*	TRINITY_DN27026_c0_g1	[EC:2.3.1.74]	4.76	Chalcone synthase	K00660
*CHS-7*	TRINITY_DN41086_c0_g1	[EC:2.3.1.74]	4.07	Chalcone synthase	K00660
*CHS-8*	TRINITY_DN44412_c0_g1	[EC:2.3.1.74]	3.9	Chalcone synthase	K00660
*CHS-1*	TRINITY_DN31744_c0_g1	[EC:2.3.1.74]	3.81	Chalcone synthase	K00660
*CHS-4*	TRINITY_DN1660_c0_g1	[EC:2.3.1.74]	3.78	Chalcone synthase	K00660
*F3*′*H-3*	TRINITY_DN11652_c0_g1	[EC:1.14.14.82]	2.58	Flavonoid 3′-monooxygenase	K05280
*F3*′*H-1*	TRINITY_DN6751_c0_g1	[EC:1.14.14.82]	1.66	Flavonoid 3′-monooxygenase	K05280
*F3*′*H-2*	TRINITY_DN12838_c0_g1	[EC:1.14.14.82]	1.4	Flavonoid 3′-monooxygenase	K05280
*CCo AOMT*	TRINITY_DN10067_c0_g1	[EC:2.1.1.104]	2.09	Caffeoyl-CoA *O*-methyltransferase	K00588
*PGT1-1*	TRINITY_DN20633_c0_g2	[EC:2.4.1.357]	1.84	Phlorizin synthase	K22845
*PGT1-2*	TRINITY_DN14776_c0_g1	[EC:2.4.1.357]	−1.25	Phlorizin synthase	K22845
*CYP73A*	TRINITY_DN514_c0_g1	[EC:1.14.14.91]	1.54	Trans-cinnamate 4-monooxygenase	K00487
*PGT1-3*	TRINITY_DN5509_c0_g1	[EC:2.4.1.357]	1.36	Phlorizin synthase	K22845
*C12RT1*	TRINITY_DN41878_c0_g1	[EC:2.4.1.236]	1.26	Flavanone 7-*O*-glucoside 2″-*O*-β-L-rhamnosyltransferase	K13080
*F3H*	TRINITY_DN5579_c0_g1	[EC:1.14.11.9]	1.01	Flavanone-3-hydroxylase	K00475
*DFR*	TRINITY_DN4510_c0_g1	[EC:1.1.1.219 1.1.1.234]	−1.29	Bifunctional dihydroflavonol 4-reductase	K13082

**Table 7:** Correlation between differential hormone and differential Anthocyanidin glycosidase Gene.

Plant Hormone	Hormone KO	Gene	Correlation
ent-7alpha-Hydroxykaur-16-en-19-oic acid	C11875	*FLS-2*	−0.998 *
ent-7alpha-Hydroxykaur-16-en-19-oic acid	C11875	*CHS-5*	−0.998 *
ent-7alpha-Hydroxykaur-16-en-19-oic acid	C11875	*CHS-6*	−0.999 *
Gibberellin A19	C02034	*CYP73A*	−0.998 *
Gibberellin A7	C11867	*PGT1-3*	0.999 *
Gibberellin A5	C11871	*F3*′*H-1*	−0.999 *
Gibberellin A5	C11871	*F3*′*H-2*	−0.997 *
Gibberellin A5	C11871	*PGT1-2*	0.998 *
Gibberellin A4	C11864	*PGT1-3*	−0.998 *
trans-Zeatin-7-β-D-glucoside	C16443	*CCo AOMT*	−0.999 *
trans-Zeatin-7-β-D-glucoside	C16443	*FLS-1*	−1.000 *
trans-Zeatin riboside monophosphate	C16430	*PGT1-3*	−1.000 *
trans-Zeatin riboside monophosphate	C16430	*C12RT1*	−0.998 *
trans-Zeatin riboside monophosphate	C16430	*DFR*	1.000 *
Isopentenyladenosine-5′-diphosphate	C16426	*C12RT1*	0.998 *
Isopentenyladenosine-5′-diphosphate	C16426	*HCT-2*	0.998 *
*O*-β-D-Xylosylzeatin	C03300	*PGT1-1*	0.998 *
*O*-β-D-Xylosylzeatin	C03300	*PGT1-3*	0.997 *
*O*-β-D-Xylosylzeatin	C03300	*C12RT1*	1.000 **
*O*-β-D-Xylosylzeatin	C03300	*DFR*	−1.000 *
Jasmonic acid	C08491	*PGT1-3*	−0.999 *
L-Methionine	C00073	*FLS-2*	−0.999 *
L-Methionine	C00073	*CHS-5*	−0.999 *
L-Methionine	C00073	*CHS-6*	−0.999 *
1-Aminocyclopropane-1-carboxylate	C01234	*CHS-4*	−0.999 *
1-Aminocyclopropane-1-carboxylate	C01234	*FLS-2*	−0.997 *
1-Aminocyclopropane-1-carboxylate	C01234	*CHS-5*	−0.998 *
1-Aminocyclopropane-1-carboxylate	C01234	*CHS-6*	−0.997 *
1-Aminocyclopropane-1-carboxylate	C01234	*CHS-7*	−1.000 *
1-Aminocyclopropane-1-carboxylate	C01234	*CHS-8*	−0.999 *
1-Aminocyclopropane-1-carboxylate	C01234	*F3* *’H-3*	−1.000 **

Note: * means *p* < 0.05, significant correlation; ** means *p* < 0.01, extremely significant correlation.

**Table 8:** Correlation between differential hormone metabolites and differential Anthocyanidin glycoside-related metabolites.

Plant Hormone	KO	Compound	Compound Abbreviation	Correlation
Gibberellin A7	C11867	Cyanidin 3-*O*-rutinoside 5-*O*-β-D-glucoside	Cy-R-B-D-G	−1.000 *
Gibberellin A7	C11867	Cyanidin 3-*O*-(6-*O*-p-coumaroyl)glucoside	Cy-*O*-P-C-G	−0.999 *
Gibberellin A7	C11867	Delphinidin	Delphinidin	−1.000 **
Gibberellin A7	C11867	Dihydrokaempferol	Dihydrokaempferol	−1.000 **
Gibberellin A9	C11863	Cyanidin 3-*O*-rutinoside 5-*O*-β-D-glucoside	Cy-R-B-D-G	−0.999 *
Gibberellin A9	C11863	Cyanidin	Cyanidin	1.000 **
Gibberellin A4	C11864	Cyanidin 3-*O*-rutinoside 5-*O*-β-D-glucoside	Cy-R-B-D-G	−1.000 **
Gibberellin A4	C11864	Delphinidin	Delphinidin	1.000 *
Gibberellin A4	C11864	Dihydrokaempferol	Dihydrokaempferol	1.000 **
trans-Zeatin-7-β-D-glucoside	C16443	Albireodelphin	Albireodelphin	−1.000 **
trans-Zeatin-7-β-D-glucoside	C16443	Pelargonidin 3-*O*-rutinoside 5-*O*-β-D-glucoside	Pel-R-B-D-G	1.000 *
trans-Zeatin-7-β-D-glucoside	C16443	Delphinidin 3-*O*-β-D-glucoside 5-*O*-(6-coumaroyl-β-D-glucoside)	Del-B-D-G-C-B-D-G	−1.000 *
trans-Zeatin-7-β-D-glucoside	C16443	Delphinidin 3-*O*-β-D-sambubioside	Del-B-D-S	1.000 **
trans-Zeatin riboside monophosphate	C16430	Pelargonidin 3-*O*-β-D-glucoside 5-*O*-(6-coumaroyl-β-D-glucoside)	Pel-B-D-G-C-B-D-G	−1.000 **
trans-Zeatin riboside monophosphate	C16430	Cyanidin 3-*O*-(6-*O*-p-coumaroyl)glucoside	Cy-*O*-P-C-G	1.000 *
trans-Zeatin riboside monophosphate	C16430	Dihydroquercetin	Dihydroquercetin	−1.000 **
trans-Zeatin riboside triphosphate	C16428	Pelargonidin 3-*O*-(6-*O*-malonyl-β-D-glucoside)	Pel-M-B-D-G	−0.999 *
trans-Zeatin riboside	C16431	Dihydrokaempferol	Dihydrokaempferol	1.000 *
Jasmonic acid	C08491	Cyanidin 3-*O*-rutinoside 5-*O*-β-D-glucoside	Cy-R-B-D-G	−0.999 *
Jasmonic acid	C08491	Delphinidin	Delphinidin	1.000 **
Jasmonic acid	C08491	Dihydrokaempferol	Dihydrokaempferol	1.000 *
Jasmonic acid	C08491	Pelargonidin 3-*O*-β-D-glucoside 5-*O*-(6-coumaroyl-β-D-glucoside)	Pel-B-D-G-C-B-D-G	−0.998 *
1-Aminocyclopropane-1-carboxylate	C01234	Albireodelphin	Albireodelphin	−0.997 *
1-Aminocyclopropane-1-carboxylate	C01234	Pelargonidin 3-*O*-rutinoside 5-*O*-β-D-glucoside	Pel-R-B-D-G	0.999 *
Isopentenyladenosine-5′-diphosphate	C16426	Leucocyanidin	Leucocyanidin	1.000 **
*O*-β-D-Xylosylzeatin	C03300	Pelargonidin 3-*O*-(6-*O*-malonyl-β-D-glucoside)	Pel-M-B-D-G	0.998 *
*O*-β-D-Xylosylzeatin	C03300	Pelargonidin 3-*O*-β-D-glucoside 5-*O*-(6-coumaroyl-β-D-glucoside)	Pel-B-D-G-C-B-D-G	0.999 *

Note: * means *p* < 0.05, significant correlation; ** means *p* < 0.01, extremely significant correlation.

**Table 9:** Transcription factor families with current 10 gene numbers.

Transcription Factor Family	Number of Genes
MYB_related	33
FAR1	32
ERF	32
C2H2	32
bHLH	28
NAC	25
GRAS	21
bZIP	19
WRKY	17
Dof	15

**Table 10:** Coexpression of transcription factors and key enzyme genes for Anthocyanidin glycoside synthesis.

Regulated Gene	TF	Gene	Correlation
TRINITY_DN221_c1_g1	BHLH130	*F3*′*H-3*	−0.954
TRINITY_DN221_c1_g1	BHLH130	*FLS-2*	1.000
TRINITY_DN10101_c0_g1	MYBCD5	*F3*′*H-3*	0.984
TRINITY_DN10101_c0_g1	MYBCD5	*HCT-2*	0.957
TRINITY_DN10101_c0_g1	MYBCD5	*CHS-2*	0.978
TRINITY_DN23487_c0_g1	MYB15	*F3*′*H-3*	−0.955
TRINITY_DN23487_c0_g1	MYB15	*FLS-2*	1.000
TRINITY_DN11681_c0_g1	MYB84	*HCT-2*	0.913
TRINITY_DN11681_c0_g1	MYB84	*F3*′*5*′*H*	−1.000
TRINITY_DN7304_c0_g1	ERF066	*F3*′*H-3*	−1.000
TRINITY_DN7304_c0_g1	ERF066	*FLS-2*	0.955
TRINITY_DN7304_c0_g1	ERF066	*CHI*	0.961
TRINITY_DN7304_c0_g1	ERF066	*CHS-3*	−0.904
TRINITY_DN7304_c0_g1	ERF066	*CHS-5*	0.954
TRINITY_DN7304_c0_g1	ERF066	*CHS-2*	−0.925
TRINITY_DN7304_c0_g1	ERF066	*F3*′*5*′*H*	0.998
TRINITY_DN13352_c0_g1	ERF5	*F3*′*H-2*	−0.937
TRINITY_DN67106_c0_g1	ERF105	*F3*′*H-2*	0.968
TRINITY_DN67106_c0_g1	ERF105	*FLS-1*	0.994
TRINITY_DN19459_c0_g1	GATA28	*FLS-2*	−0.998
TRINITY_DN19459_c0_g1	GATA28	*F3*′*H-3*	0.936
TRINITY_DN19459_c0_g1	GATA28	*CHI*	−0.997
TRINITY_DN19459_c0_g1	GATA28	*HCT-1*	−0.914
TRINITY_DN19459_c0_g1	GATA28	*F3*′*5*′*H*	−0.913
TRINITY_DN19459_c0_g1	GATA28	*F3*′*H-2*	−0.994
TRINITY_DN6935_c0_g1	GATA24	*FLS-1*	−0.968
TRINITY_DN63776_c0_g1	GATA16	*FLS-1*	−0.971
TRINITY_DN2067_c1_g1	TCP19	*DFR*	0.999
TRINITY_DN13320_c0_g1	bZIP21	*F3*′*H-1*	0.986
TRINITY_DN15659_c0_g1	ERF1	*CHS-5*	0.987
TRINITY_DN15659_c0_g1	ERF1	*HCT-1*	0.952
TRINITY_DN15659_c0_g1	ERF1	*F3*′*H-2*	−0.994
TRINITY_DN6935_c0_g1	GATA24	*FLS-1*	−0.968

**Table 11:** Promoter elements of Anthocyanidin glycoside related transcription factors.

Transcription Factor	Hormone-Acting Element	Number	Hormone
BHLH130	CGTCA-motif	2	Me_JA
BHLH130	G-box	7	Me_JA
BHLH130	ABRE	7	ABA
BHLH130	TCA-element	1	SA
ERF1	ABRE	5	ABA
ERF1	TCA-element	2	SA
ERF1	TATC-box	2	GA
ERF1	CGTCA-motif	1	Me_JA
ERF1	G-box	5	Me_JA
ERF5	ABRE	2	ABA
ERF5	G-box	4	Me_JA
ERF066	CGTCA-motif	1	Me_JA
ERF066	G-Box	8	Me_JA
ERF066	ABRE	7	ABA
ERF066	TCA-element	1	SA
ERF105	CGTCA-motif	1	Me_JA
ERF105	TCA-element	1	SA
ERF105	TATC-box	1	GA
ERF105	AuxRR-core	1	AUX
GATA16	CGTCA-motif	2	Me_JA
GATA16	TCA-element	2	SA
GATA16	TGA-box	1	AUX
GATA24	CGTCA-motif	1	Me_JA
GATA24	ABRE	3	ABA
GATA24	TCA-element	2	SA
GATA24	AuxRR-core	1	AUX
GATA24	G-box	3	Me_JA
GATA28	CGTCA-motif	2	Me_JA
GATA28	TCA-element	1	SA
GATA28	TATC-box	1	GA
MYB15	CGTCA-motif	1	Me_JA
MYB15	ABRE	2	ABA
MYB15	TCA-element	2	SA
MYB15	G-box	2	Me_JA
MYB84	CGTCA-motif	1	Me_JA
MYB84	P-box	1	GA
MYBCD5	TCA-element	1	SA
MYBCD5	P-box	1	GA

**Table 12:** Correlation between endogenous hormones and Anthocyanidin glycoside-related transcription factors.

Plant Hormone	Transcription Factor	Correlation
ent-7alpha-Hydroxykaur-16-en-19-oic acid	BHLH130	−0.998 *
ent-7alpha-Hydroxykaur-16-en-19-oic acid	MYB15	−0.998 *
Gibberellin A53	BHLH74	−0.997 *
Gibberellin A24	MYBCD5	0.999 *
Gibberellin A24	GATA1	0.998 *
Gibberellin A20	MYBCD5	1.000 **
Gibberellin A20	GATA1	1.000 **
Gibberellin A8	GATA24	−0.999 *
Gibberellin A44 diacid	BHLH130	0.999 *
Gibberellin A44 diacid	MYB15	1.000 *
Gibberellin A34	BHLH74	0.998 *
Gibberellin A34	BHLH78	0.997 *
Salicylic acid	ERF5	0.999 *
Dihydrozeatin	BHLH74	0.997 *
Zeatin	ERF12	−1.000 **
trans-Zeatin-7-β-D-glucoside	BHLH8	0.999 *
trans-Zeatin-7-β-D-glucoside	BHLH74	1.000 *
trans-Zeatin-7-β-D-glucoside	BHLH78	1.000 *
trans-Zeatin riboside monophosphate	TCP19	1.000 **
trans-Zeatin riboside monophosphate	bZIP21	0.998 *
Isopentenyladenosine-5’-diphosphate	ERF5	−0.999 *
*O*-β-D-Glucosylzeatin	BHLH8	1.000 *
*O*-β-D-Glucosylzeatin	BHLH74	0.999 *
*O*-β-D-Glucosylzeatin	BHLH78	0.999 *
*O*-β-D-Glucosylzeatin	ERF105	−1.000 **
Dihydrozeatin-*O*-glucoside	BHLH8	1.000 *
Dihydrozeatin-*O*-glucoside	BHLH74	1.000 **
Dihydrozeatin-*O*-glucoside	BHLH78	1.000 **
Dihydrozeatin-*O*-glucoside	ERF105	−0.998 *
trans-Zeatin riboside triphosphate	TCP19	0.998 *
trans-Zeatin riboside triphosphate	bZIP21	1.000 **
*O*-β-D-Xylosylzeatin	TCP19	−0.999 *
*O*-β-D-Xylosylzeatin	bZIP21	−1.000 *
(-)-Jasmonoyl-L-isoleucine	ERF12	1.000 **
Jasmonic acid	TCP19	0.998 *
L-Methionine	BHLH130	−0.999 *
L-Methionine	MYB15	−0.999 *
1-Aminocyclopropane-1-carboxylate	BHLH130	−0.998 *
1-Aminocyclopropane-1-carboxylate	GATA28	1.000 **
1-Aminocyclopropane-1-carboxylate	MYB15	−0.997 *

Note: * means *p* < 0.05, significant correlation; ** means *p* < 0.01, extremely significant correlation.

## Data Availability

The data have been registered in the NCBI Sequence Read Archive (SRA) database (https://trace.ncbi.nlm.nih.gov/Traces/sra/, accessed on 22 June 2024) under the GenBank accession numbers SRR29496896, SRR29496895, SRR29496894, SRR29496893, SRR29496892, SRR29496891, SRR29496890, SRR29496889, SRR29496888, SRR29496887, SRR29496886, and SRR29496885.

## Supplementary Materials

The following supporting information can be downloaded at: https://www.mdpi.com/article/10.3390/genes15080989/s1, The electropherograms presenting the RNA bands in agarose gels (Figures S1–S9) and the RNA integrity number (Figure S10) were in the Supplementary Materials. Real-time quantitative PCR verification: the GenBank accession of the reference gene is JF825424. The amplification and melt curves are shown in the Supplementary Materials (Figures S11–S28).
