# Supplementary figures and images for: Comparative Metabolome and Transcriptome Analyses of the Regulatory Mechanism of Light Intensity in the Synthesis of Endogenous Hormones and Anthocyanins in Anoectochilus roxburghii (Wall.) Lindl
Source: Genes (Basel). 2024 Jul 26;15(8):989. doi: 10.3390/genes15080989 (PMC11353614; doi:10.3390/genes15080989)

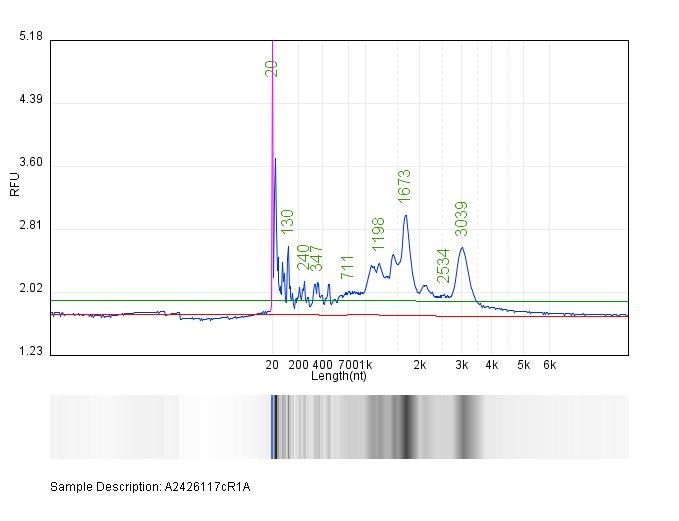

Supplement: Supplementary file 1 [file genes-15-00989-s001.zip › Figure S1 The electropherograms presenting the RNA bands in agarose gels of T1A.jpg]

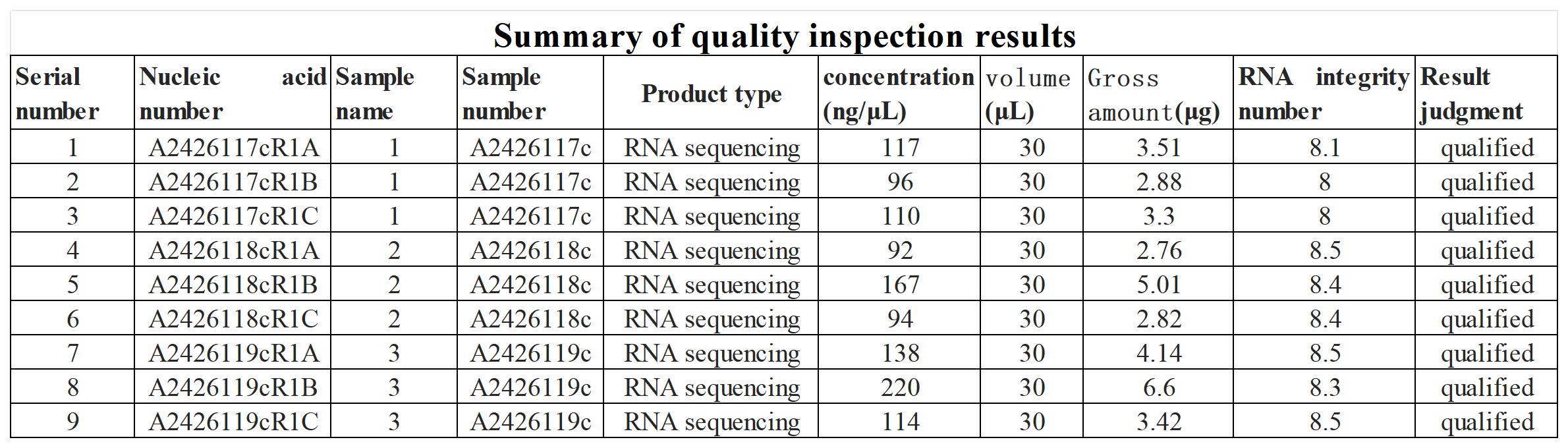

Supplement: Supplementary file 1 [file genes-15-00989-s001.zip › Figure S10 RNA integrity number.jpg]

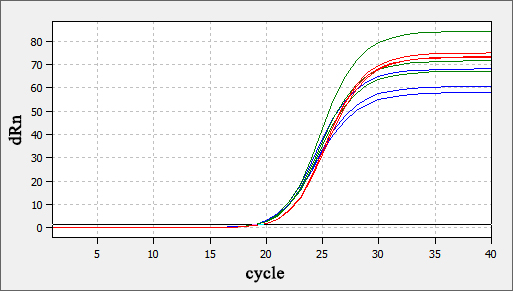

Supplement: Supplementary file 1 [file genes-15-00989-s001.zip › Figure S11 gene actin,amplification curve.jpg]

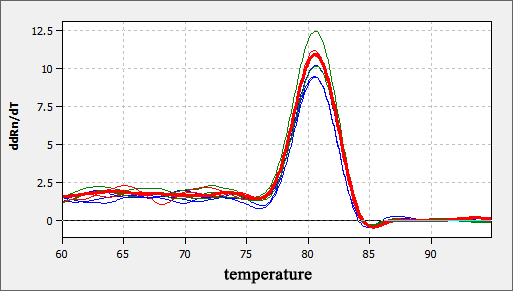

Supplement: Supplementary file 1 [file genes-15-00989-s001.zip › Figure S12gene actin,melting curve.jpg]

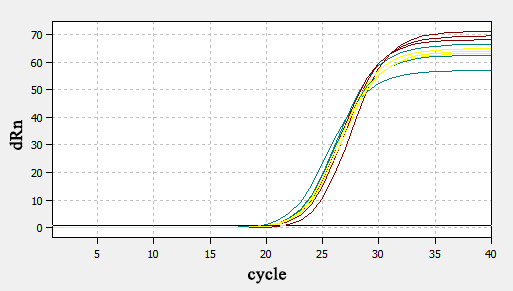

Supplement: Supplementary file 1 [file genes-15-00989-s001.zip › Figure S13gene FLS-2,amplification curve.jpg]

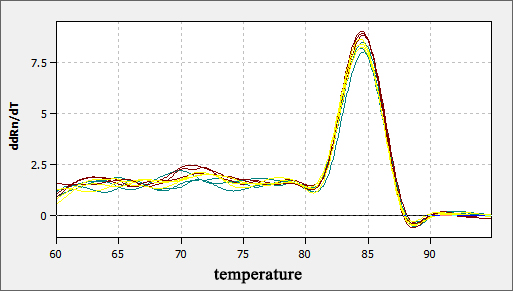

Supplement: Supplementary file 1 [file genes-15-00989-s001.zip › Figure S14gene FLS-2,melting curve.jpg]

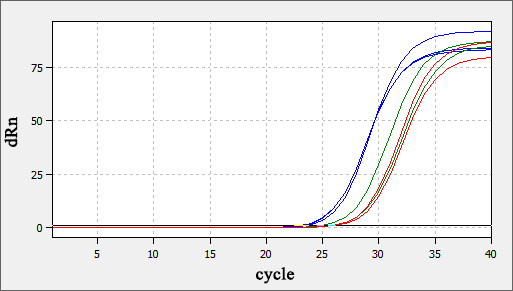

Supplement: Supplementary file 1 [file genes-15-00989-s001.zip › Figure S15gene CHS-3,amplification curve.jpg]

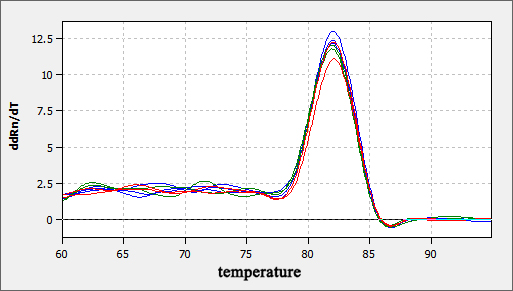

Supplement: Supplementary file 1 [file genes-15-00989-s001.zip › Figure S16gene CHS-3,melting curve.jpg]

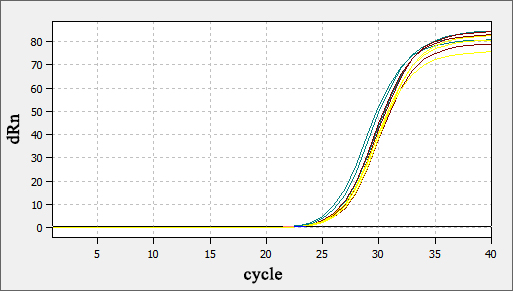

Supplement: Supplementary file 1 [file genes-15-00989-s001.zip › Figure S17gene CHS-4,amplification curve.jpg]

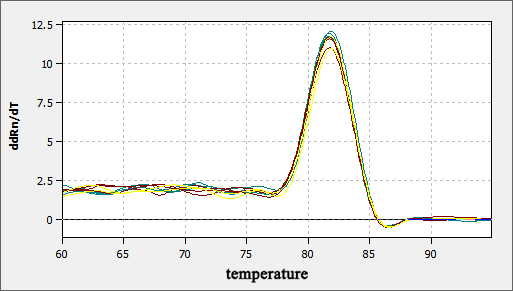

Supplement: Supplementary file 1 [file genes-15-00989-s001.zip › Figure S18gene CHS-4,melting curve.jpg]

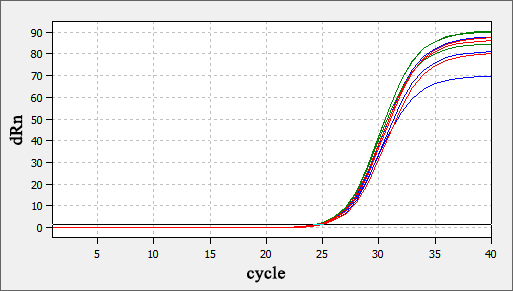

Supplement: Supplementary file 1 [file genes-15-00989-s001.zip › Figure S19gene CHS-7,amplification curve.jpg]

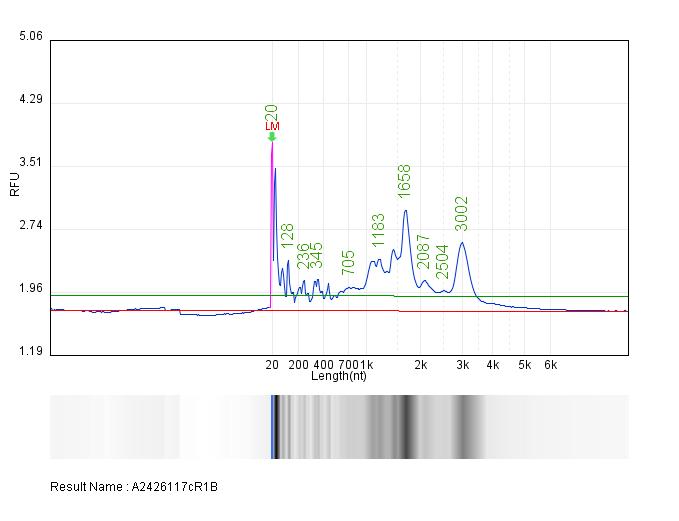

Supplement: Supplementary file 1 [file genes-15-00989-s001.zip › Figure S2 The electropherograms presenting the RNA bands in agarose gels of T1B.jpg]

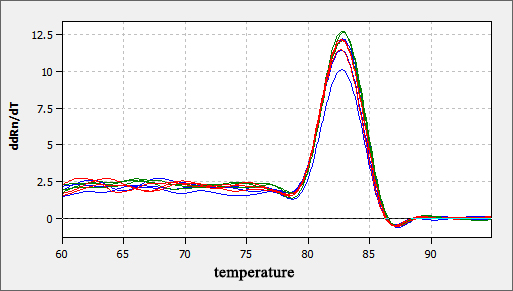

Supplement: Supplementary file 1 [file genes-15-00989-s001.zip › Figure S20gene CHS-7,melting curve.jpg]

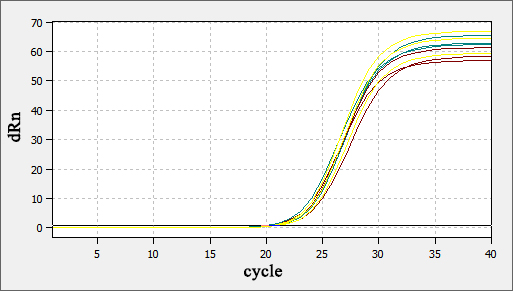

Supplement: Supplementary file 1 [file genes-15-00989-s001.zip › Figure S21gene F3'H-3,amplification curve.jpg]

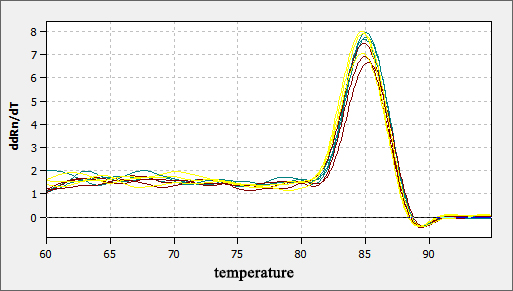

Supplement: Supplementary file 1 [file genes-15-00989-s001.zip › Figure S22gene F3'H-3,melting curve.jpg]

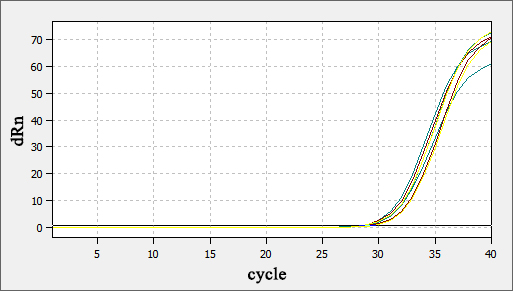

Supplement: Supplementary file 1 [file genes-15-00989-s001.zip › Figure S23gene DFR,amplification curve.jpg]

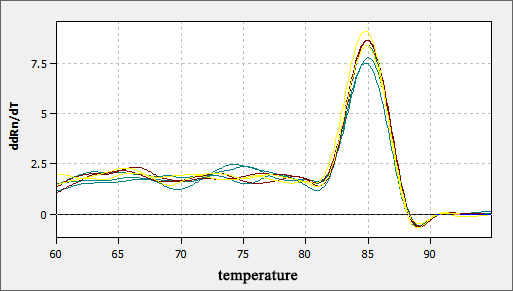

Supplement: Supplementary file 1 [file genes-15-00989-s001.zip › Figure S24gene DFR,melting curve.jpg]

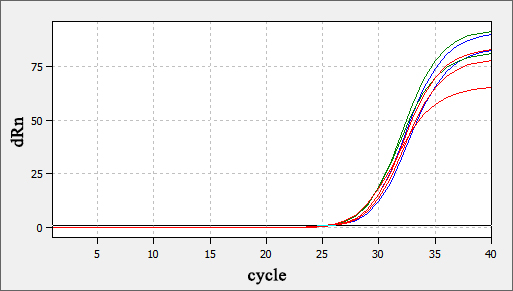

Supplement: Supplementary file 1 [file genes-15-00989-s001.zip › Figure S25 Transcription factor bHLH130,amplification curve.jpg]

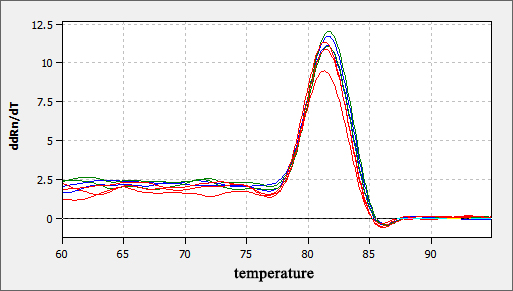

Supplement: Supplementary file 1 [file genes-15-00989-s001.zip › Figure S26 Transcription factor bHLH130,melting curve.jpg]

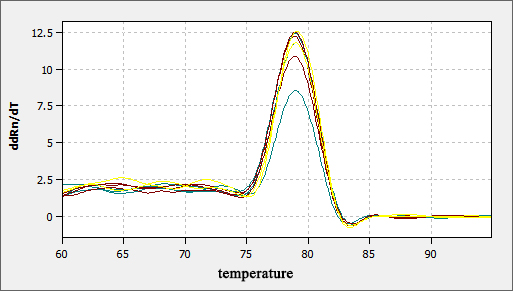

Supplement: Supplementary file 1 [file genes-15-00989-s001.zip › Figure S27Transcription factor ERF066, melting curve.jpg]

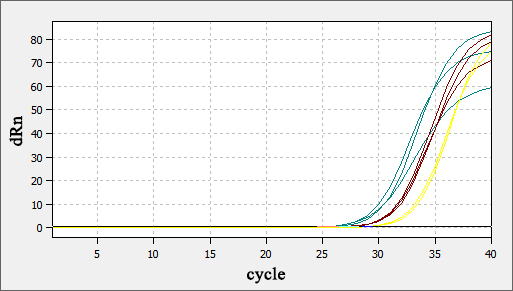

Supplement: Supplementary file 1 [file genes-15-00989-s001.zip › Figure S28 Transcription factor ERF066,amplification curve.jpg]

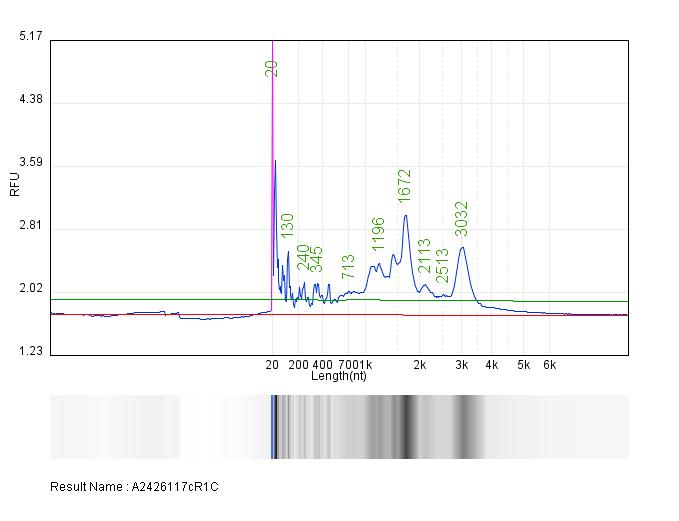

Supplement: Supplementary file 1 [file genes-15-00989-s001.zip › Figure S3 The electropherograms presenting the RNA bands in agarose gels of T1C.jpg]

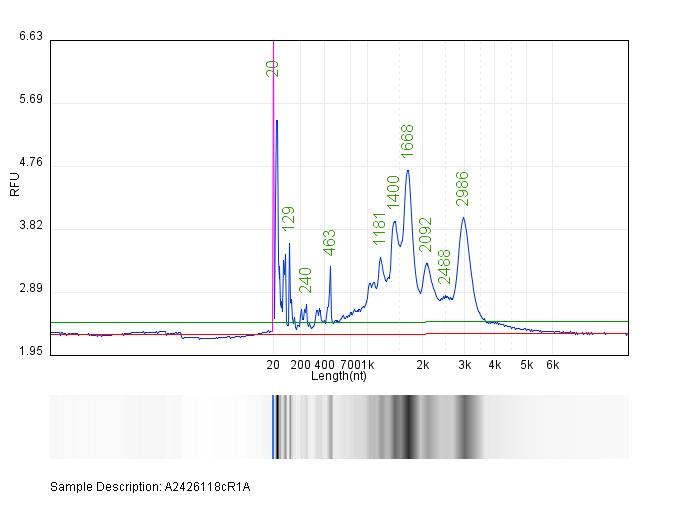

Supplement: Supplementary file 1 [file genes-15-00989-s001.zip › Figure S4 The electropherograms presenting the RNA bands in agarose gels of T2A.jpg]

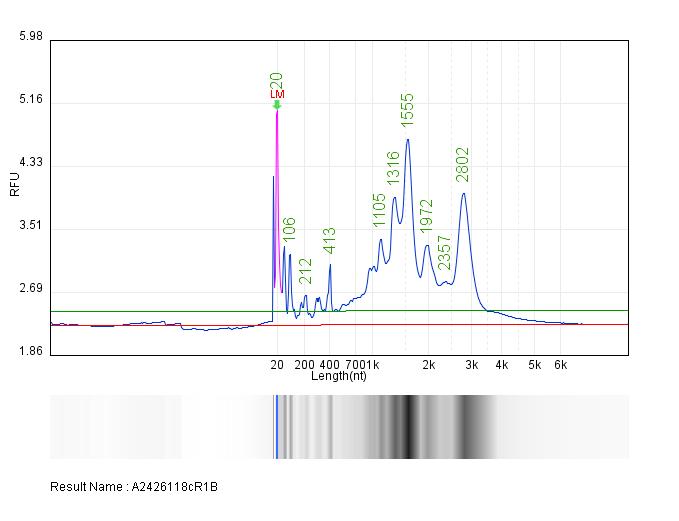

Supplement: Supplementary file 1 [file genes-15-00989-s001.zip › Figure S5 The electropherograms presenting the RNA bands in agarose gels of T2B.jpg]

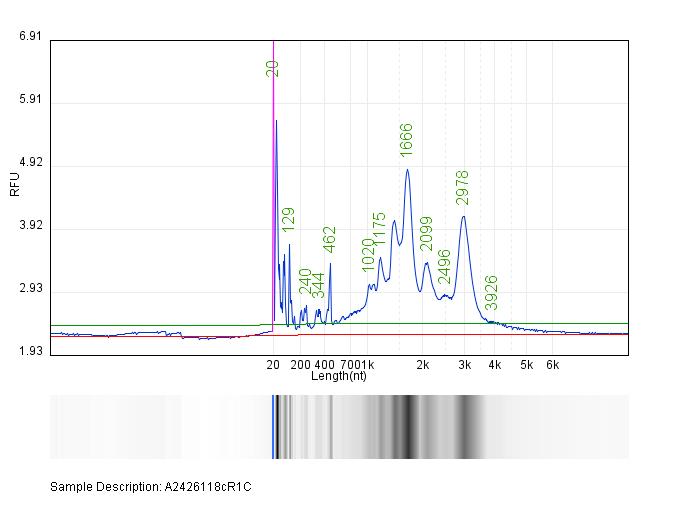

Supplement: Supplementary file 1 [file genes-15-00989-s001.zip › Figure S6 The electropherograms presenting the RNA bands in agarose gels of T2C.jpg]

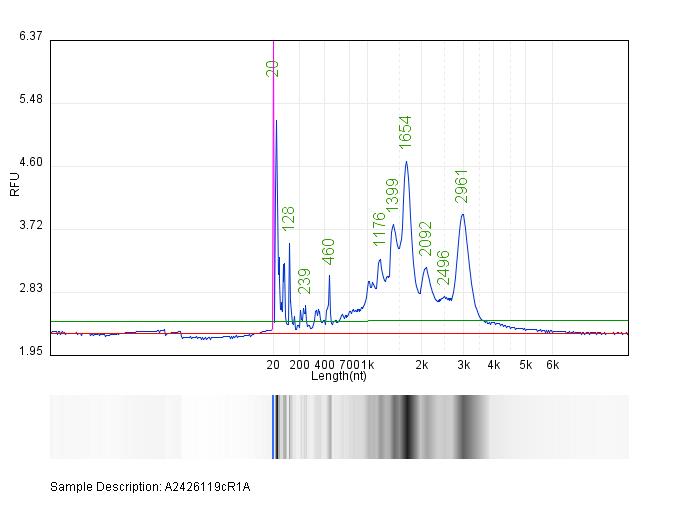

Supplement: Supplementary file 1 [file genes-15-00989-s001.zip › Figure S7 The electropherograms presenting the RNA bands in agarose gels of T3A.jpg]

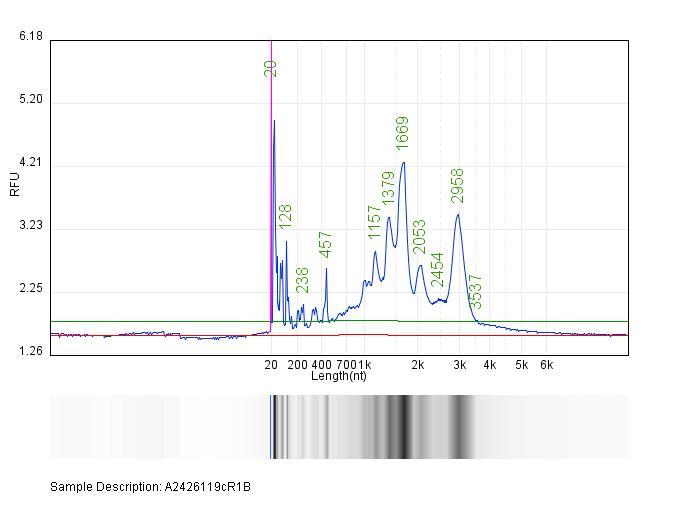

Supplement: Supplementary file 1 [file genes-15-00989-s001.zip › Figure S8 The electropherograms presenting the RNA bands in agarose gels of T3B.jpg]

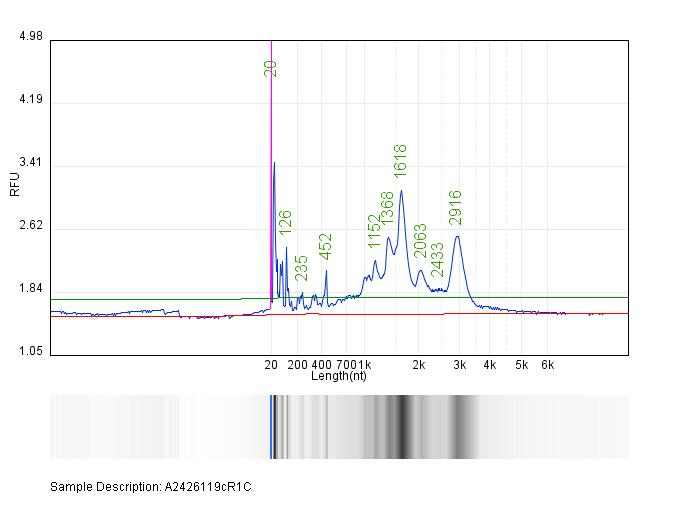

Supplement: Supplementary file 1 [file genes-15-00989-s001.zip › Figure S9 The electropherograms presenting the RNA bands in agarose gels of T3C.jpg]
